# The Bovine Metabolome

**DOI:** 10.3390/metabo10060233

**Published:** 2020-06-05

**Authors:** Aidin Foroutan, Carolyn Fitzsimmons, Rupasri Mandal, Hamed Piri-Moghadam, Jiamin Zheng, AnChi Guo, Carin Li, Le Luo Guan, David S. Wishart

**Affiliations:** 1 Department of Agricultural, Food and Nutritional Science, University of Alberta, Edmonton, AB T6G 2P5, Canada; aidin@ualberta.ca (A.F.); cfitzsim@ualberta.ca (C.F.); lguan@ualberta.ca (L.L.G.); 2 Department of Biological Sciences, University of Alberta, Edmonton, AB T6G 2E9, Canada; rmandal@ualberta.ca (R.M.); pirimogh@ualberta.ca (H.P.-M.); jiamin3@ualberta.ca (J.Z.); aguo@ualberta.ca (A.G.); cbli@ualberta.ca (C.L.); 3 Agriculture and Agri-Food Canada, Edmonton, AB T6G 2P5, Canada; 4 Department of Computing Science, University of Alberta, Edmonton, AB T6G 2E8, Canada

**Keywords:** bovine, tissue, biofluid, NMR, LC–MS/MS, ICP–MS, metabolite

## Abstract

From an animal health perspective, relatively little is known about the typical or healthy ranges of concentrations for many metabolites in bovine biofluids and tissues. Here, we describe the results of a comprehensive, quantitative metabolomic characterization of six bovine biofluids and tissues, including serum, ruminal fluid, liver, *Longissimus thoracis* (LT) muscle, semimembranosus (SM) muscle, and testis tissues. Using nuclear magnetic resonance (NMR) spectroscopy, liquid chromatography–tandem mass spectrometry (LC–MS/MS), and inductively coupled plasma–mass spectrometry (ICP–MS), we were able to identify and quantify more than 145 metabolites in each of these biofluids/tissues. Combining these results with previous work done by our team on other bovine biofluids, as well as previously published literature values for other bovine tissues and biofluids, we were able to generate quantitative reference concentration data for 2100 unique metabolites across five different bovine biofluids and seven different tissues. These experimental data were combined with computer-aided, genome-scale metabolite inference techniques to add another 48,628 unique metabolites that are biochemically expected to be in bovine tissues or biofluids. Altogether, 51,801 unique metabolites were identified in this study. Detailed information on these 51,801 unique metabolites has been placed in a publicly available database called the Bovine Metabolome Database.

## 1. Introduction

The cattle industry is among the most significant agri-food sectors in the world. It has been estimated that the global beef industry is worth more than CAD 300 billion/year [1] and is responsible for producing and processing > 70 million tonnes/year of meat [2]. The global dairy industry is worth > CAD 650 billion/year [3] and produces more than 800 million tonnes/year of milk or milk products [4], with more than 80% of those products coming from dairy cows [5]. Beef or beef products as well as milk and milk products are rich and dense sources of vital nutrients. They are nutritionally important food staples for hundreds of millions of people around the world. Indeed, the widespread use of bovine milk and bovine meat has been responsible for significant improvements to human health, prosperity and longevity over the past 200 years [6,7,8,9,10,11]. While the macronutrient (protein, fat, etc.) content of beef and milk is well known and has been studied for many decades, somewhat less is known about the micronutrient and chemical composition of key bovine biofluids and tissues [8,12,13]. Furthermore, from an animal health perspective, even less is known about the typical or healthy ranges of concentrations for many clinically important metabolites in bovine biofluids and tissues [14,15,16,17]. Indeed, far less is known about the metabolome of dairy and beef cattle than the metabolomes of other organisms, such as humans [18,19,20,21], yeast [22,23], bacteria [24,25] or even common crop plants, such as rice or tomatoes [26,27,28].

Over the past ten years, our knowledge gaps regarding the bovine metabolome have been slowly filled in through a variety of metabolomics studies focused on characterizing the chemical composition of different bovine biofluids and tissues. In particular, our laboratory has contributed significantly to the current state of knowledge of the bovine metabolome. Our first study, published in 2013, focused on the characterization of the bovine ruminal metabolome [12]. This work, which used a combination of nuclear magnetic resonance (NMR) spectroscopy, inductively coupled plasma–mass spectrometry (ICP–MS), gas chromatography–mass spectrometry (GC–MS), and reverse-phase liquid chromatography–mass spectrometry (RPLC–MS) coupled with direct flow injection (DFI)–mass spectrometry (DFI–MS) (RPLC–DFI–MS/MS), led to the characterization and quantification of 246 ruminal fluid metabolites or metabolite species, including amino acids, biogenic amines, carbohydrates, lipids, vitamins, and trace minerals. Here, we define “metabolite species” as those molecules with non-unique chemical formulas or masses (such as lipid isomers-PC(36:6)) while “unique compound structures” correspond to compounds with a unique and clearly defined chemical structure and a unique chemical name (such as L-alanine). Subsequent studies done by our lab have explored the metabolite composition of bovine serum, ruminal fluid, and urine, leading to the identification of 142 metabolites in serum [14,15,16,17], 232 metabolites in ruminal fluid [12,29], and 52 metabolites in urine [17] using NMR, GC–MS, liquid chromatography–mass spectrometry (LC–MS) and/or ICP–MS. Most recently, we completed the most extensive metabolomics study ever done on bovine milk [13]. This study used a combination of NMR spectroscopy, LC–MS, and ICP–MS to identify and quantify 296 bovine milk metabolites or metabolite species (corresponding to 1447 unique structures). A further literature review identified 676 milk metabolites or metabolite species (corresponding to 908 unique structures), bringing the total to 972 metabolites or metabolite species [13]. Many other bovine metabolome studies have also been undertaken and published by other laboratories around the world [7,30,31,32,33,34,35]. To date there have been 45 metabolomics studies performed on bovine milk, 42 metabolomics studies on bovine serum/plasma, 25 metabolomics studies performed on bovine ruminal fluid, 4 metabolomics studies on bovine urine and more than 20 studies performed on a variety of bovine tissues, secreta and biofluids including liver, muscle, testes, and others [8,12,13,29,36,37].

While the number of metabolomics studies on bovine fluids and tissues has grown significantly, the majority of the studies published to date have focused on identifying rather than quantifying metabolites. Furthermore, most of these bovine-oriented metabolomics studies do not use more than two analytical techniques nor do they attempt to integrate previously published metabolite information to extend or validate their results. An additional challenge facing bovine metabolomics researchers lies in the fact that most of the metabolomics data is not consolidated or centralized into readily accessible public resource. To facilitate further research into the metabolome of beef and dairy cattle, we believe it is critical to create an open, publicly accessible resource that contains current, comprehensive and quantitative data on the bovine metabolome—including metabolomics data on multiple biofluids, excreta and tissues. We also believe that this resource should include detailed compound descriptions, information on referential LC–MS, GC–MS and NMR spectra, detailed biochemical pathway data and other data typically found in referential metabolome databases (refer to the Human Metabolome Database (HMDB) [18,19,20,21], the *Escherichia coli* Metabolome Database (ECMDB) [24,25], and the Yeast Metabolome Database (YMDB) [22,23]). Such an undertaking would benefit beef and dairy researchers, food scientists, nutritionists and consumers. 

To create such a resource, we decided to combine experimental metabolomics techniques with computer-aided text mining to compile essentially all of the known chemical compounds (endogenous and exogenous) that can be detected in bovine milk, blood, urine, ruminal fluid, muscle, liver, and testes (as well as other biofluids and tissues) along with their respective concentrations. The resulting database is called the Bovine Metabolome Database (BMDB). The BMDB, which is housed at www.bovinedb.ca [38], is a comprehensive web-accessible database containing concentration data, physico-chemical data and reference data for 51,801 bovine metabolites.

This paper describes the experimental, computational and literature research efforts used to collect and validate the metabolomics data in the BMDB as well as the techniques used to construct the BMDB and place it online. Experimentally, we focused on updating or expanding the data for several incompletely characterized biofluids or tissues, including bovine serum, ruminal fluid, liver, muscle, and testes. To do so, we used multiple quantitative metabolomics techniques, including high-resolution NMR spectroscopy, liquid chromatography–tandem mass spectrometry (LC–MS/MS) and ICP–MS methods. This experimental work allowed us to greatly add to the previously collected metabolomics data for these biofluids and tissues. To further enhance our experimental metabolic profiling studies, we conducted an extensive literature survey and extracted additional metabolite data from more than 240 journal articles identified through computer-aided literature searches spanning not only these biofluids/tissues but another six bovine biofluids and tissues. This “bibliomic” effort yielded data for another 1086 metabolites or metabolite species (which corresponds to 163 lipids and 923 non-lipids). The experimentally acquired metabolite data was then combined with genome-scale metabolite inference—a technique commonly used to fill in the metabolic “holes” for other metabolomes, such as the human metabolome [18,19,20,21], the yeast metabolome [22,23], and the *E. coli* metabolome [24,25]. This method uses known, organism-specific metabolic pathways [39] and known, organism-specific gene/protein reactions to provide data on metabolites that are known to exist, but not normally measured via NMR or MS techniques. This led to the addition of another 48,628 metabolites or metabolite species. All of these data (metabolite names, structures, descriptions, concentration data, biofluid/tissue locations, physico-chemical data, and known or predicted NMR and MS spectra) were then placed in the BMDB. The BMDB is a web-accessible, MySQL-based database constructed using a Ruby-on-Rails web framework. The BMDB offers a variety of user-friendly data search, browse and display options similar to other popular metabolomics databases, such as the HMDB [18,19,20,21], YMDB [22,23], and ECMDB [24,25].

Overall, the intent of this study is to help address four key questions: (1) What kinds of compounds and nutrients are present in various bovine biofluids and tissues? (2) What is the approximate variation in the concentration of metabolites across different kinds of bovine biofluids and tissues? (3) What fraction of the bovine biofluids and tissues metabolome can be identified and/or quantified using targeted, quantitative metabolomics techniques? (4) What analytical methods (NMR, LC–MS/MS, ICP–MS) are best suited for comprehensively profiling the bovine metabolome? Answering these questions will provide a common foundation and a more appropriate set of reference values for both ongoing and future bovine biofluids and tissues composition studies. 

## 2. Results

This section is divided into four subsections covering: (1) experimental metabolomics results; (2) literature review results; (3) a comparative assessment between different tissues, biofluids, and platforms; and (4) a detailed description of the BMDB.

### 2.1. Water-Soluble Compound Identification and Quantification by NMR and LC–MS/MS

Using a combination of NMR and LC–MS/MS, a total of 58, 64, 60, 60, 66, and 69 water-soluble compounds were identified and quantified in serum, ruminal fluid, *Longissimus thoracis* (LT) muscle, semimembranosus (SM) muscle, liver, and testis tissues, respectively. The complete list of compound concentrations (including averages and their standard deviations) for serum is shown in Table 1. The most abundant compounds in serum were lactate (average concentration: 4.8 ± 2 mM), glucose (4.0 ± 0.4 mM), and urea (1.3 ± 0.3 mM). The lowest concentration that could be reliably detected in serum was 0.04 ± 0.02 µM for putrescine. The complete list of compound concentrations for ruminal fluid (including averages and their standard deviations) is shown in Supplementary Table S1. The most abundant compounds in ruminal fluid were acetate (37 ± 8 mM), butyrate (26 ± 9 mM), propionate (16 ± 5 mM), and glucose (16 ± 11 mM). The lowest concentration in ruminal fluid that could be reliably detected was 0.1 ± 0.1 µM for serotonin. 

In terms of LT and SM muscle tissues, the complete list of compound concentrations (including averages and their standard deviations) is shown in Supplementary Table S2. The most abundant compounds detected in these muscle tissues, in terms of µmol per gram wet-weight were lactate (LT = 31 ± 8 µmol/g, SM = 38 ± 11 µmol/g), carnosine (LT = 22 ± 5 µmol/g, SM= 22 ± 3 µmol/g), creatine (LT = 4.7 ± 0.4 µmol/g, SM= 4.8 ± 0.3 µmol/g), and glutamine (LT = 2.8 ± 0.8 µmol/g, SM = 2.5 ± 0.7 µmol/g). The lowest concentration that could be reliably detected was for spermidine (0.09 ± 0.03 nmol/g in LT muscle and 0.11 ± 0.04 nmol/g in SM muscle) as well as spermine (0.2 ± 0.1 nmol/g in LT muscle and 0.11 ± 0.03 nmol/g in SM muscle).

Two other non-muscle tissues were analyzed, including liver and testes. The complete list of compound concentrations (including averages and their standard deviations) for liver tissue is shown in Supplementary Table S3. The most abundant compounds in liver, in terms of µmol per gram wet-weight, were glucose (80 ± 15 µmol/g), lactate (12 ± 1.7 µmol/g), and sn-glycero-3-phosphocholine (11 ± 1.5 µmol/g), while the least abundant compound was putrescine (0.2 ± 0.1 nmol/g). The complete list of compound concentrations (including averages and their standard deviations) for testis tissue is shown in Supplementary Table S4. The most abundant compounds in testis, in terms of µmol per gram wet-weight were lactate (7.7 ± 1.6 µmol/g), creatine (7.6 ± 1.9 µmol/g), myo-inositol (7.2 ± 1.2 µmol/g), O-phosphoethanolamine (7 ± 1.2 µmol/g), and glutamate (3.3 ± 0.7 µmol/g), whereas the least abundant compound was spermidine (0.4 ± 0.2 nmol/g). 

### 2.2. Lipid-Like Compound Identification and Quantification by LC–MS/MS

A locally developed LC–MS/MS assay called The Metabolomics Innovation Centre (TMIC) Prime assay provided quantitative results for 74 lipid-like compounds (14 lysophosphatidylcholines (LysoPCs), 5 sphingomyelins (SMs), 5 hydroxysphingomyelins (SM(OH)s, 10 phosphatidylcholines (PCs), and 40 acylcarnitines) for serum (Table 1), LT and SM muscles (Supplementary Table S2), liver (Supplementary Table S3), and testis (Supplementary Table S4). Sixty-four of these lipid-like compounds (10 LysoPCs, 3 SMs, 3 SM(OH)s, 8 PCs, and 40 acylcarnitines) were detected and quantified in ruminal fluid (Supplementary Table S1). The other 10 compounds that could be quantified in serum and tissue samples, but not in ruminal fluid, were below the limit of detection (LOD) in ruminal fluid samples. Note that each LysoPC and PC species identified by the TMIC Prime assay typically corresponds to a minimum of two up to 24 possible unique structures, respectively. In our study, SM(16:0) (68 ± 10 µM) and LysoPC(14:0) (6 ± 3 µM) were the most abundant lipids identified in serum and ruminal fluid, respectively. Carnitine or C0 (1856 ± 424 nmol/g and 1751 ± 337 nmol/g) was the most abundant metabolite identified in LT and SM muscle, respectively. Likewise, carnitine (22 ± 6 nmol/g) and SM(16:0) (22 ± 7 nmol/g) were the most abundant lipid-like compounds identified by the TMIC Prime assay in liver, whereas SM(16:0) (42 ± 6 nmol/g) was the most abundant lipid-like compound in testis. The least abundant lipids detected by TMIC Prime assay were acylcarnitines. These included C16:2-OH (5 ± 1 nM) and C18:2 (6 ± 1 nM) in serum and ruminal fluid, respectively. They also included a single acylcarnitine, C18:2, at 0.004 ± 0.001 nmol/g, 0.004 ± 0.001 nmol/g, 0.006 ± 0.001 nmol/g, and 0.004 ± 0.001 nmol/g for LT muscle, SM muscle, liver, and testis, respectively. 

### 2.3. Trace Element Identification and Quantification by ICP–MS

The ICP–MS method was developed to detect and quantify 35 metabolites or trace minerals. While no toxic trace metals (i.e., Pb, As, Cd) were detected in the samples, ICP–MS provided quantitative results for 13 metabolites or trace minerals in bovine serum (Table 1). The most abundant trace elements identified and quantified by ICP–MS were sodium (134 ± 14 mM), potassium (4 ± 0.4 mM), calcium (2 ± 0.2 mM), and phosphorus (1.3 ± 0.2 mM), while the least abundant metals quantified by ICP–MS were cesium (1.7 ± 0.3 nM), barium (200 ± 30 nM), and strontium (1000 ± 100 nM). In terms of ruminal fluid, our ICP–MS analysis provided quantitative results for 17 metabolites or trace minerals, as shown in Supplementary Table S1. The most abundant trace elements identified and quantified were sodium (236 ± 20 mM), potassium (40 ± 7 mM), phosphorus (12 ± 2 mM), and magnesium (7.5 ± 3.5 mM), whereas the least abundant compounds were cesium (30 ± 10 nM) and cobalt (1000 ± 200 nM). 

ICP–MS could identify and quantify 15, 14, and 15 trace minerals in bovine LT muscle, SM muscle, and liver, respectively (Supplementary Tables S2 and S3). In LT and SM muscles, the most abundant trace elements identified and quantified were potassium (LT = 49 ± 8 µmol/g; SM = 52 ± 7 µmol/g), phosphorus (LT = 22 ± 3 µmol/g; SM = 22 ± 3 µmol/g), and sodium (LT = 10 ± 2 µmol/g; SM = 12 ± 3 µmol/g), whereas the least abundant compounds were thallium (LT= 0.0007 ± 0.0002 nmol/g; SM= 0.0009 ± 0.0001 nmol/g) and vanadium (LT = 0.013 ± 0.004 nmol/g; SM= 0.009 ± 0.001 nmol/g). In liver, the most abundant trace elements identified and quantified were potassium (46 ± 8 µmol/g), phosphorus (33 ± 7 µmol/g), and sodium (26 ± 5 µmol/g), while the least abundant compounds were cesium (0.02 ± 0.01 nmol/g), and lead (0.019 ± 0.003 nmol/g). For testis tissue, ICP–MS could identify and quantify 16 metabolites or trace minerals (Supplementary Table S4). The most abundant trace elements identified and quantified were potassium (49 ± 8 µmol/g), sodium (36 ± 7 µmol/g), and phosphorus (16 ± 3 µmol/g). The least abundant compounds were thallium (0.0014 ± 0.0003 nmol/g) and lead (0.014 ± 0.003 nmol/g).

### 2.4. The Chemical Composition of Bovine Biofluids and Tissues (Experimental Data)

Inspection of our experimental data reveals that the chemical composition of bovine serum is dominated by inorganic ions (primarily sodium, potassium, calcium, and phosphorus), carbohydrates (glucose), organic acids (lactate, acetate, and 3-hydroxybutyrate), amino acids (glycine, valine, and glutamine), and various amine-containing compounds (urea, creatinine). Ruminal fluid mostly contains inorganic ions (primarily sodium, potassium, phosphorus, and magnesium), carbohydrates (glucose), organic acids (acetate, butyrate, and propionate), amino acids (lysine, glutamate, and alanine), biogenic amines (methylamine and putrescine), and nucleobases (uracil). The LT and SM muscles are mostly composed of inorganic ions (primarily potassium, phosphorus, and sodium), carbohydrates (glucose), organic acids (lactate), amino acids (creatine, glutamine, and alanine), biogenic amines (carnosine) and miscellaneous compounds (O-acetylcarnitine and betaine). The most abundant compounds in liver are inorganic ions (primarily potassium, phosphorus, and sodium), carbohydrates (glucose), organic acids (lactate), amino acids (glutamate and glycine), biogenic amines (taurine) and miscellaneous compounds (sn-Glycero-3-phosphocholine). The most abundant chemicals in testis are inorganic ions (primarily potassium, sodium, and phosphorus), carbohydrates (UDP-N-acetylglucosamine), organic acids (lactate), amino acids (creatine and glutamate), biogenic amines (O-phosphoethanolamine and taurine), and miscellaneous polyols (myo-inositol). Lesser quantities of lipids including acylcarnitines (except carnitine and acetylcarnitine, which have high concentrations in both LT and SM muscles), LysoPCs, PCs, SMs, as well as other small bioactive compounds are also evident in these bovine biofluids and tissues. 

### 2.5. Literature Survey of Bovine Biofluids and Tissues Metabolites

As part of our literature survey, 249 papers were reviewed on bovine metabolomics. These papers provided metabolomic data for six biofluids and eight tissues. The paper that provided the most extensive metabolomics data was written by Foroutan et al. [13] on the milk metabolome. This paper identified and quantified 972 metabolites (296 metabolites were experimentally detected and 676 were found from the literature) found in bovine milk using a combination of LC–MS/MS, NMR, ICP–MS and literature reviews [13]. Other papers of note include the work of Saleem et al. [12], which described more than 200 metabolites found in bovine ruminal fluid (using a combination of NMR, GC–MS, ICP–MS, and LC–MS/MS), the paper by Zhang et al. [16], which identified and quantified 128 metabolites found in serum (using a combination of direct injection and tandem mass spectrometry (DI-MS/MS) with a reverse-phase LC–MS/MS), and the paper by Muroya et al. [8], which identified and quantified 70 metabolites found in LT muscle of Japanese Black (Wagyu) cattle (using Capillary Electrophoresis–Time-of-flight–Mass Spectrometry, also known as CE–TOF–MS). The most thoroughly studied bovine biofluid was milk with 118 papers, followed by blood with 35 papers [36]. The least studied bovine biofluid was semen with three papers [54,55,56]. The most thoroughly studied bovine tissue was muscle with 12 papers [7,8,36,57,58], whereas the least studied bovine tissue was liver, with just three papers [59,60,61]. The most comprehensively characterized bovine biofluid was milk with 972 metabolites identified via literature data [13], followed by ruminal fluid with ~ 200 metabolites [12]. 

As we observed with both our experimental data and from the literature data, bovine serum is particularly rich in inorganic ions or minerals such as sodium (107–137 mM), potassium (4–4.3 mM), calcium (1.4–2.2 mM), phosphorus (1.3–1.6 mM), magnesium (0.8–0.9 mM), as well as a variety of organic acids such as lactate (0.6–1.6 mM), acetate (0.9–1 mM), and 3-hydroxybutyrate (0.2–2 mM). Other highly abundant metabolites reported in bovine serum include glucose (3–4 mM), urea (2–4 mM), and acetone (0.1–1 mM). The least abundant compounds in serum include several acylcarnitines, such as C18:1-OH (8–9 nM), C16-OH (3–6 nM), as well as C3-OH and C7-DC and C14 (10–12 nM). The concentrations reported in the literature data for the above-mentioned metabolites agreed well with our experimental data, except for lactate (2.8–6.8 mM), acetate (0.2–0.6 mM), and urea (1.1–1.6 mM).

Just as with serum, bovine ruminal fluid is rich in a variety of inorganic ions or minerals, such as sodium (110–117 mM), potassium (17.9–18.2 mM), phosphorus (9.1–9.3 mM), calcium (0.9–1 mM), and magnesium (0.09–0.1 mM). The most abundant organic compounds reported in ruminal fluid are organic acids such as acetate (41–81 mM), propionate (14–17 mM), and butyrate (6–18 mM), amino acids such as proline (240–1275 µM), isoleucine (123–1210 µM), and lysine (91–1095 µM), as well as glucose (393–3111 µM). The least abundant compounds in ruminal fluid include several lipids or acylcarnitines such as C16:2 (2–13 nM), C18:1-OH (2–22 nM), C14:2-OH (8–32 nM), and C16-OH (9–11 nM), sphingomyelins such as SM(16:0) (10–50 nM), hydroxysphingomyelins such as SM (16:1(OH)) (10–20 nM) and SM (14:1(OH)) (10–30 nM), as well as the lysophosphatidylcholine, LysoPC16:0 (10–200 nM). The concentrations reported in the literature for the above-mentioned metabolites agreed well with our experimental data, except for sodium (216–255 mM), potassium (33.3–46.5 mM), phosphorus (10.5–14.3 mM), magnesium (3.9–10.9 mM), glucose (4746–27122 µM), SM(16:0) (130–1370 nM), SM(16:1(OH)) (30–110 nM), and SM(14:1(OH)) (40–60 nM). These differences are most likely due to dietary differences among the different cattle groups being studied, as the concentration of many ruminal fluid metabolites are strongly affected by the feed given to cattle. 

In terms of bovine LT and SM muscles, the literature data indicated that organic acids such as lactate (14–132 µmol/g), inosinate (0.031–9 µmol/g), and succinate (0.9–2.3 µmol/g), amino acids such as creatine (1–10 µmol/g), glutamine (3–5 µmol/g), and alanine (1–1.3 µmol/g), carbohydrates such as glucose (3.3 µmol/g) and glucose-1-phosphate (0.1–1.2 µmol/g), the biogenic amine, carnosine (10–14 µmol/g), as well as betaine (1.5–1.8 µmol/g) and carnitine (0.8–2.6 µmol/g) were found to be quite abundant in these tissues. The least abundant compounds in LT and SM muscles are several amino acids such as aspartate (14–54 nmol/g) and tryptophan (35–95 nmol/g), as well as the biogenic amine, putrescine (9–22 nmol/g). The concentrations reported in the literature for the above-mentioned metabolites agreed with our experimental data, except for glucose (LT: 0.3–0.8 µmol/g, SM: 0.3–1.2 µmol/g) and carnosine (LT: 17–27 µmol/g, SM: 19–25 µmol/g). 

Regarding bovine liver tissue, a single study described by Miles et al. [59] was the only study that attempted to identify and quantify metabolites in the bovine liver tissue. The most abundant compound found was glutamate (6122–7999 nmol/g), followed by alanine (2366–3515 nmol/g) and glutamine (1911–2576 nmol/g). The least abundant compounds found in liver in this study was ornithine (984–1184 nmol/g). These values agreed moderately well with our findings.

We found literature values for 12 amino acids in bovine testis tissue. The most abundant metabolites reported in the literature for bovine testis are glutamate (2–2.4 µmol/g), glycine (0.9–1.3 µmol/g), and alanine (0.9–1.2 µmol/g), while the least abundant compounds are histidine (20 nmol/g), isoleucine (30 nmol/g), leucine (40 nmol/g), valine (50 nmol/g), and lysine (40–200 nmol/g). These values agreed moderately well with our findings with the observed differences likely due to diet, age and breed, chemical volatility, sample work-up or extraction, sample storage protocols, and instrument sensitivity.

### 2.6. The BMDB Website

**Figure 1:**
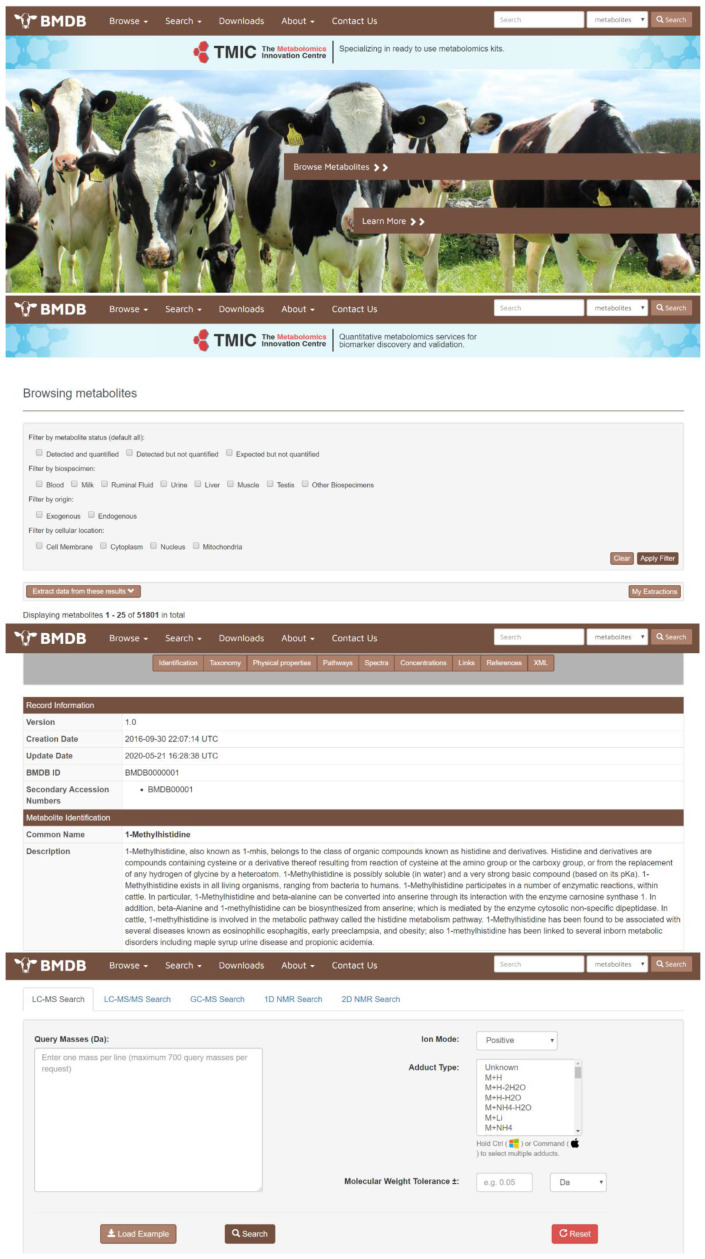
Screenshot montage of different browsing and searching screens taken from the Bovine Metabolome Database (BMDB). A more detailed description of the different functions and capabilities of the various browse and search tools in the BMDB is given in the text.

All of the detected, identified, quantified and biochemically inferred compounds obtained from our experimental, computational, and literature-searching efforts have been deposited into a freely accessible database called the BMDB (Bovine Metabolome Database—www.bovinedb.ca). The database contains not only the metabolite names and synonyms (common and International Union of Pure and Applied Chemistry (IUPAC)), but also their structures (multiple formats), basic descriptions, chemical ontology, physico-chemical properties, their reference spectra (NMR, GC–MS, and LC–MS), pathway information (as derived from PathBank) [39], and literature citations from the scientific literature for all (to the best of our knowledge) of these compounds. The BMDB is structured to be very similar to other popular, online metabolomics databases, such as the HMDB [18,19,20,21], ECMDB [24,25] or YMDB [22,23]. In particular, the BMDB is designed to serve as a user-friendly tool for data browsing and compound searching. The BMDB can be launched from its home page (Figure 1) using a variety of pull-down menus or tabs located at the top of the home page, which serve as a navigation panel. Users may “Browse”, “Search”, or “Downloads” data from the database using this navigation panel. Additional information about the database is located under the “About” menu. Clicking on the “Browse” tab and the subsequent “Metabolites” dropdown option (on the BMDB navigation panel) generates a tabular view that allows users to casually scroll through the metabolites in the database or to re-sort its contents by compound name or mass. Each compound entry in the BMDB is hyperlinked to an individual metabolite description table (called a MetaboCard) that, when clicked, brings up additional information on that particular chemical. Clicking on the “Search” tab at the top of the home page allows users to perform compound name searches, general text searches, compound structure searches, molecular weight searches, and a variety of spectral (NMR and MS) searches. The “Downloads” section contains several data files that include the chemical structures, metabolite data, spectra, and other files (in SDF, CSV, or XML format). Currently, the BMDB contains information on 3173 unique, experimentally detected compounds with unique, well-defined structures and names. The BMDB also contains another 48,628 compounds that have been computationally inferred to exist from detailed genomic analysis, biochemical pathway analysis, and comparison to other well-studied mammalian metabolomes. As a result, the BMDB contains three classes of metabolites: (1) detected and quantified; (2) detected but not quantified; and (3) expected (or genomically/biochemically inferred). These classes of metabolites may be easily selected or filtered using BMDB’s filtering functions.

Note that the total number of chemical compounds in BMDB is not a number that will remain unchanged. Certainly, as technology and instrument sensitivity improve, it is anticipated that this number will increase as other, lower abundance metabolites will be detected and will be added to future versions of the BMDB.

## 3. Discussion

Using a combination of experimental, computational and literature-based approaches, we have attempted to identify and quantify as many chemicals as possible that are detectable in different biofluids and tissues of both dairy and beef cattle. We have deposited this information into the BMDB (www.bovinedb.ca) [38]. The BMDB contains a total of 51,801 metabolites with unique compound structures. Just 4.1% of these metabolites with unique structures (corresponding to 11.8% metabolites or metabolite species) have concentration data in at least one tissue or biofluid, while the remaining 95.9% of these metabolites with unique structures (or 88.2% metabolites or metabolite species) have no quantification information whatsoever. The metabolites in the BMDB have been associated with eight bovine tissues and six different bovine biofluids. Of the 2100 compounds with unique structures that were experimentally identified and quantified in the BMDB, 1834 (87.3%) were experimentally characterized by our laboratory (using NMR, GC–MS, LC–MS and ICP–MS methods) over the past 7 years, including 306 (14.6%), as reported in this communication. Another 266 (12.7%) metabolites were compiled from computer-aided literature searches and were therefore measured by other laboratories. In addition, 48,628 (93.9%) metabolites were biochemically inferred to exist through detailed comparisons to existing genomics/metabolomics data of other well-studied mammals.

There is considerable variability in the extent of metabolome coverage for different bovine tissues and biofluids. These results are summarized in Table 2. This table shows that the most fully characterized bovine biofluid is milk with 928 known metabolites or metabolite species (corresponding to 2350 unique structures). The most poorly characterized biofluid is urine with 62 known metabolites or metabolite species (corresponding to 62 unique structures). Likewise, the most completely characterized bovine tissue is liver with 1056 known metabolites or metabolite species (corresponding to 1254 unique structures) while the most poorly characterized tissues or biofluids are parathyroid gland, pineal gland, umbilical cord, and vitreous humor. These poorly characterized tissues are grouped in Table 2 under the “All tissues” category, each with just one known metabolite (corresponding to one unique structure). 

Analysis of the data in the BMDB also allows us to identify which metabolomics platforms are most useful or most widely used for bovine metabolomics studies. According to our data, the most popular metabolomics platform for analyzing bovine metabolites is NMR, with 26 published studies. GC–MS is a distant second with 13 published studies, while LC–MS was used for 12 studies. ICP–MS has been used with just three studies. Other platforms used by metabolomics researchers include GC×GC–MS with 11 studies, and high-performance liquid chromatography–ultraviolet spectroscopy (HPLC–UV) with eight studies. In terms of metabolome coverage, LC–MS methods have been used to identify 472 compounds and to quantify 154 compounds. On the other hand, NMR methods have been used to identify 170 compounds and to quantify 108 compounds. GC–MS has been used to identify 425 compounds and quantify 41 compounds, while ICP–MS has been used to identify and quantify 44 compounds. Based on the list of chemicals identified by each platform there appears to be considerable overlap with the compounds identified by NMR and the compounds identified by GC–MS. On the other hand, LC–MS tends to permit the identification and quantification of more hydrophobic compounds. As mentioned earlier, the differences in metabolite coverage from the different platform technologies are largely due to differences in sensitivity, detection/instrument biases and separation protocols, as well as differences in compound stability, solubility, volatility and other intrinsic chemical factors [13]. 

### 3.1. Comparisons to Other Studies

One of the objectives of this work was to evaluate the level of agreement between different platforms and different laboratories (i.e., protocols) in identifying and quantifying key metabolites across identical biofluids and tissues and over different periods of time. Overall, we found a very good agreement between the results reported for most methods and most laboratories. Here, we define a very good agreement as being within one standard deviation of the reported literature value (i.e., literature value ± 1 SD). Moderate agreement corresponds to being within two standard deviations of the reported value, while a poor agreement is defined as a value that is greater than two standard deviations of the reported literature value. A small number of questionable or profoundly different, literature-derived concentration values were identified during our literature survey, but most of these could be eliminated through the curation process after being deemed mistaken, disproven (by subsequently published studies), mistyped or physiologically impossible. Much of the curation process involved multiple curators carefully reading and double-checking the primary literature to annotate the concentration unit types, to perform unit conversions, and to catch typographical inconsistencies.

In terms of serum composition, we found that almost all studies exhibited very good agreement across platforms and across laboratories with almost no significant differences in either the composition or concentration of reported metabolites. More specifically, in terms of water-soluble compounds, an inspection of Table 1 reveals a generally good agreement between the NMR- and LC–MS-measured concentrations and those reported in the literature. Forty-one out of the 58 compounds identified by these two techniques in serum had concentration values previously reported in the literature. More than 73% (30/41) of these compounds exhibited good agreement with literature values (i.e., the average values from our experiments fell within one standard deviation of the literature value) in serum. ICP–MS is considered as a gold standard for the identification and quantification of trace metal ions [62], and, therefore, we tend to have higher confidence in the values derived via ICP–MS over those measured by other (older or less sensitive) technologies. In serum, all nine trace elements identified and quantified in our study exhibited very good agreement with previously reported literature values. In total, 64 out of the 145 compounds identified by NMR, LC–MS/MS, and ICP–MS had concentration values that were previously reported in the literature. More than 78% (50/64) of these compounds exhibited very good agreement with literature values for bovine serum. For instance, the value of alanine reported by our study ranged from 210–270 µM, and for the literature-derived data it ranged from 151–222 µM. This widespread agreement was not unexpected as serum/plasma must be highly stable and cannot vary much in its metabolite concentrations to ensure physiological homeostasis [63]. Of course, there were a few exceptions to this rule. The most variable metabolite reported in serum was betaine. The value of betaine reported by our study ranged from 138–200 µM, and the literature-reported values ranged from 14–26 µM. This variation could be due to a number of factors, including differences in diet, sex, age, breed, sample work-up or extraction, sample storage protocols, analytical platforms and instrument sensitivity.

In contrast to serum and plasma, ruminal fluid exhibited considerable variability across platforms and laboratories, even after normalizing to creatinine. In terms of water-soluble compounds, the inspection of Supplementary Table S1 reveals a generally good agreement between the NMR- and LC–MS-measured concentrations and those reported in the literature. A total of 55 out of the 64 compounds identified in ruminal fluid had concentration values previously reported in the literature. More than 65% (36/55) of these compounds exhibited good agreement with literature values (i.e., the average values from our experiments fell within one standard deviation of the literature value) in ruminal fluid. Regarding trace elements, 15 out of the 17 the compounds identified in our study had concentration values previously reported in the literature. Five of the 17 compounds exhibited good agreement with literature values (i.e., the average values from our experiments fell within one standard deviation of the literature value). In total, 93 out of the 145 compounds identified by NMR, LC–MS/MS, and ICP–MS in the ruminal fluid had concentration values previously reported in the literature. More than 58% (54/93) of these compounds exhibited good agreement with literature values (i.e., the average values from our experiments fell within one standard deviation of the literature value) in ruminal fluid. 

It was of some interest to compare our earlier ruminal fluid analysis done in 2013 [12] with the more recent analysis reported here. The most variable metabolites reported in ruminal fluid were 3-phenylpropionate and magnesium. The value of 3-phenylpropionate reported by our 2020 study ranged from 0.03–0.07 mM, while the value reported in our 2013 study ranged from 0.3–0.7 mM. We re-analyzed our 2013 data and found a graphical error in fitting the 3-phenylpropionate’s chemical shifts in NMR, which explains the concentration difference between these two studies. The value of magnesium reported by our 2020 study ranged from 4–11 mM, while the value reported in our 2013 study ranged from 0.09–0.10 µM. We speculate that the differences in the concentration of magnesium is mainly due to the dietary treatment differences in this study versus our earlier 2013 study. 

In terms of tissues, we found generally good agreement between the results obtained for the metabolite composition of muscle, testis, and liver. For water-soluble compounds in muscle tissues, an inspection of Supplementary Table S2 reveals a generally good agreement between the NMR- and LC–MS-measured concentrations and those reported in the literature. A total of 43 out of the 60 compounds identified in both LT and SM muscles had concentration values previously reported in the literature. For the trace minerals identified in our study, we could not find any values reported in the literature. In total, 43 out of the 149 as well as 43 out of 148 compounds identified by NMR, LC–MS/MS, and ICP–MS in the LT and SM muscles, respectively, had concentration values previously reported in the literature. More than 65% (28/43) of these compounds exhibited very good agreement with literature values (i.e., the average values from our experiments fell within one standard deviation of the literature value) in muscle tissues. For instance, the value of beta-alanine reported for NMR ranged from 92–206 µM in LT muscle and 85–159 µM in SM muscle, whereas the literature-derived values ranged from 84–155 µM. The most variable compounds seen in muscle tissues were carnosine (LT: 17–27 µmol/g, SM: 19–25 µmol/g, as compared to Muroya et al. [8]: 10–14 µmol/g) and glucose (LT: 0.3–0.8 µmol/g, SM: 0.3–1.2 µmol/g, as compared to Kim et al. [57]: 3.3 µmol/g). We suspect that these variations may be due to factors such as differences in diet, sex, age, breed, chemical volatility, sample work-up or extraction, sample storage protocols, and instrument sensitivity. Similarly, 12 out of the 159 compounds (12 out of 69 water-soluble compounds) as well as 4 out of 155 compounds (4 out of 66 water-soluble compounds) identified in the testis and liver tissues, respectively, had concentration values previously reported in the literature. Forty-one percent (5/12) of the compounds found in testis tissue exhibited very good agreement with literature values (i.e., the average values from our experiments fell within one standard deviation of the literature value). However, the magnitude of this variation was small in testis. As with other tissues, we suspect these small variations may be due to the same factors, such as differences in diet, sex, age, etc., mentioned above.

Interestingly, none of the four metabolites previously reported in liver (including alanine, glutamate, glutamine, and ornithine) exhibited very good agreement with the values we measured experimentally, although the magnitude of this variation was small. The reasons for this appear to be due to differences in sample processing and the fact that the values reported by Miles et al. [59] were measured via an HPLC coupled with a fluorometric detection instrument. Furthermore, a comparison of these values reported by Miles et al. [59] to metabolite values found in other tissues and other mammals [64,65,66] suggests that their values are likely incorrect. 

### 3.2. Comparisons Across Platforms

We also found that, for those metabolites that were measured by both LC–MS/MS and NMR, there was a generally good overall agreement with the concentration values. Depending on the sample type, NMR and LC–MS/MS were able to identify a common set of 20–26 metabolites. Table 3 lists the concentrations measured by NMR and by LC–MS/MS for these common metabolites in the serum analyzed in this study. As can be seen from this table, the values differed by less than 10%, on average. The most significant exceptions were for sarcosine (28%) and glucose (14%) with the concentrations reported by NMR being 4 µM for sarcosine and 4.6 mM for glucose, whereas the concentrations reported by LC–MS/MS were 3 µM for sarcosine and 4 mM for glucose. Regarding sarcosine, the values are at the lower limit of sensitivity for NMR and so the differences are likely due to instrumental noise. However, glucose has 13 peaks (chemical shifts) in the NMR spectrum where some of these peaks overlap with other compounds (i.e., aspartate and taurine). As a result, these spectral overlaps can affect the concentration of glucose measured by NMR. Furthermore, these differences are likely a consequence of small differences in sample preparation or separation protocols, as well as differences in compound stability, solubility, volatility and other intrinsic chemical factors [13]. 

The metabolome coverage we achieved was maximized by using as many different platforms as possible and carefully optimizing the metabolite coverage of each platform. Some platforms clearly performed better than others. Depending on the type of sample, 39–49 compounds could be identified and quantified using NMR spectroscopy whereas 13–17 metal ions could be identified and quantified by ICP–MS. On the other hand, LC–MS/MS identified and quantified 102–116 compounds (depending on the sample type) of which 38–42 were non-lipid compounds and 64–74 were lipid or lipid-like compounds. 

Because of their fundamentally different separation and detection technologies, different metabolomics platforms tend to target or detect different classes of metabolites. For instance, NMR is relatively “untargeted” but is biased towards highly abundant, water-soluble compounds. Other methods were quite targeted, with ICP–MS being limited only to metal ions and LC–MS/MS (the TMIC Prime assay) being limited to a pre-selected set of 143 compounds, including amino acids, biogenic amines, organic acids, and lipid-like compounds. While this study did employ a relatively wide range of metabolomics platforms (NMR, LC–MS/MS, ICP–MS), it did not use all available detection tools (GC–MS, GC×GC-TOF), nor did it explore all available separation protocols (e.g., solid phase extraction and enrichment, immune or ELISA detection, chemical derivatization, etc.). However, for this particular study, we wanted to address the question of how well a cross section of commonly accessible metabolomics technologies or platforms could perform in identifying and quantifying metabolites in various bovine biofluids and tissues. 

Overall, LC–MS/MS appears to be the most suitable method for the characterization of bovine biofluids and tissues metabolites, especially in terms of its broad coverage (primarily of lipids) and its amenability to quantification. It also requires very little sample volume (10 µL), is relatively inexpensive (on a per sample basis), largely automated, and offers a high-throughput route for measuring metabolites. The other methods, such as NMR and ICP–MS offer complementary data to LC–MS/MS. However, they do not provide the breadth of coverage, the level of automation or the sensitivity available via LC–MS/MS. 

## 4. Materials and Methods 

This study consisted of both “wet” (experimental) and “dry” (computational/literature) research. The “wet” component focused on the comprehensive, quantitative metabolomics characterization of six bovine biofluids and tissues using multiple metabolomics platforms. The “dry” component consisted of computer-aided literature research to complement and complete the collection of metabolomics data on the eight selected biofluids/tissues as well as four other bovine biofluids and tissues. It also included computational, genome-scale inference of biochemically expected bovine metabolites. The dry component also consisted of designing, constructing and testing the electronic BDMB database. This section will describe the materials and methods for both the wet and dry components of the study. 

### 4.1. Ethics Approvals

The collection and analysis of bovine tissues and biofluids in this study were approved by the University of Alberta’s Animal Care Committee (Animal Use Protocol (AUP) 1129) under the auspices of the Canadian Council of Animal Care [67]. 

### 4.2. Animal Selection

Twenty-six purebred Angus bulls raised on the Roy Berg, University of Alberta Kinsella Ranch (Kinsella, AB, Canada), were used in this study. After weaning, the bulls were fed and managed according to industry standards for feedlot production of finished cattle in Alberta until they reached approximately 17 months of age [68]. The bulls were then slaughtered at the Agriculture and Agri-Food Canada (AAFC)-Lacombe Research Centre abattoir over a period of four days to collect the target tissues [68]. 

### 4.3. Sample Collection

Two bovine biofluids including serum and ruminal fluid, and four bovine tissues including liver, LT muscle, SM muscle, and testis tissues were collected and experimentally characterized for this study. Serum and ruminal fluids were collected from live animals, whereas the tissues were collected from recently slaughtered animals. Blood samples (10 mL) were collected in the morning (just before feeding) at 15 months of age from a jugular vein using vacutainer serum collection tubes (Becton Dickinson, Mississauga, ON, Canada). Blood samples were kept in a cooler on ice, transferred to the laboratory within 3 hours after collection, and centrifuged at 2000× *g* at 4 °C for 15 min. The upper layer of serum was then collected, and 4 mL was stored at −80 °C. Ruminal fluid samples were collected at 9 months of age through a rumen oro-ruminal tube in the morning prior to feeding. A 15 mL sample of ruminal fluid for each animal was obtained and placed on ice, then centrifuged at 3000× *g* for 20 min at 4 °C to remove particular matter. The supernatant was then transferred into a 5 mL tube and centrifuged at 9400× *g* for 20 min at 4 °C for further phase separation. The clear upper phase from each tube was transferred into two 2 mL Eppendorf tubes and subsequently stored at −80 °C. To collect tissue samples (muscle, liver, and testes), the animals were slaughtered at age 17 months. Between 5 and 10 g tissue samples from LT muscle (from the left side of the animal between the 12th and 13th ribs), SM muscle (left), liver, and testes were collected approximately 30–45 min after death. The tissue was immediately frozen in liquid nitrogen and then stored at −80 °C. 

### 4.4. Biofluid Sample Preparation for NMR

Biofluid sample preparation was done according to the procedure described by Foroutan et al. [69]. Both serum and ruminal fluid samples contain a substantial portion of large molecular weight proteins and lipoproteins which can seriously compromise the quality of ^1^H-NMR spectra though the generation of intense, broad lines that interfere with the identification and quantification of lower abundance metabolites. De-proteinization can eliminate these peaks. The de-proteinization of these samples was done by ultrafiltration using 3-kDa ultrafiltration units (Amicon Micoron YM-3; Sigma-Aldrich, St. Louis, MO, USA), following a previously reported de-proteinization procedure [70]. Briefly, a newly opened 3 kDa Amicon 0.5 mL ultrafilter system was thoroughly rinsed by adding 500 µL HPLC-grade water (Millipore Sigma, Oakville, ON, Canada) to the filter and centrifuging at 9400× *g* for 10 min. The glycerol-containing filtrate was disposed into the sink followed by repeating the rinsing step four more times. The rinsed Amicon ultrafilter was then dried with a Kimwipe tissue and placed into a new 1.5 mL microcentrifuge tube. The biofluid sample (either ruminal fluid or serum), which was thawed on ice (for 30 min), was briefly centrifuged at 9400× *g* for 3 min at 4 °C to remove any particulate material. The supernatant (450 µL) was then transferred into the Amicon ultrafilter and centrifuged at 11,500× *g* for 20 min at 4 °C to remove the proteins from the sample. The de-proteinized sample was then frozen and stored at −80 °C until further use.

### 4.5. Tissue Sample Preparation for NMR

For each tissue, approximately 2 g of a frozen sample was ground in a 300 mL pestle and mortar with about 100 mL liquid nitrogen. A total of 300 mg of the ground (still frozen) tissue was weighed and transferred into an 11 mL PYREX™ screw cap glass tube (Corning Inc., New York, NY, USA), followed by adding 4.4 mL cold methanol and 0.68 mL cold HPLC grade water. The tissue was homogenized using a Vortex-Genie 2 vortexer (Scientific Industries, New York, NY, USA) at 1500 rpm for 3 min, then 2.2 mL cold chloroform was added to the sample. The mixture was vortexed at 1500 rpm speed for 5 min and then centrifuged for 10 min at 1000× *g* at 4 °C. The supernatant was then transferred into a new 11 mL PYREX™ glass tube, then 2.2 mL cold chloroform and 3.2 mL cold water were added. The mixture was vortexed at 1500 rpm speed for 3 min, then centrifuged for 10 min at 1000× *g* at 4 °C. This will give a biphasic mixture. The upper polar phase (containing water-soluble metabolites) and lower non-polar phase (containing lipid-soluble metabolites) were carefully separated using a Pasteur pipette. The lower non-polar phase was transferred into a 2 mL PYREX™ glass tube and kept at −80 °C for future metabolomics analysis. The upper polar phase was transferred into a 15 mL Falcon tube (Thermo Fisher Scientific, Whitby, ON, Canada). The upper polar phase was purged under nitrogen gas for 90–120 min. Once the purging was completed, 2 mL of HPLC-grade water was added to the tube. The tube was then frozen with liquid nitrogen and the sample lyophilized for 24 h. After lyophilization, the dried sample was dissolved with 300 µL of HPLC-grade water and kept at −80 °C until further use.

### 4.6. NMR Spectroscopy

Two-hundred-eighty μL of the ultrafiltered biofluid (serum or ruminal fluid) or the water-soluble extract of each tissue was transferred to a 1.5 mL Eppendorf tube, to which an additional 70 μL of a standard NMR buffer solution was added. For serum, the buffer consisted of 250 mM potassium phosphate (pH 7.0), 5 mM 2,2-dimethyl-2-silapentane-5 sulfonate (DSS-d_6_), 5.84 mM 2-chloropyrimidine-5-carboxylic acid, and D_2_O 54% v/v in H_2_O. For ruminal fluid, liver, SM and LT muscle and testis tissue extracts, the buffer consisted of 750 mM potassium phosphate (pH 7.0), 5 mM 2,2-dimethyl-2-silapentane-5 sulfonate (DSS-d_6_), 5.84 mM 2-chloropyrimidine-5-carboxylic acid, and D_2_O 54% v/v in H_2_O. The mixture (a final volume of 350 μL) was then transferred to a 3 mm NMR tube for spectral analysis. All ^1^H-NMR spectra were collected on a Bruker Avance III Ascend 700 MHz spectrometer equipped with a 5 mm cryo-probe (Bruker Biospin, Rheinstetten, Germany). ^1^H-NMR spectra were collected at 25 °C using the first transient of a nuclear Overhauser effect spectroscopy (NOESY)-presaturation pulse sequence. This pulse sequence was selected based on its excellent reproducibility and quantitative accuracy [71]. NMR spectra were acquired with 128 scans employing a 4 second acquisition time and a 1 second recycle delay.

#### NMR Compound Identification and Quantification 

Prior to spectral deconvolution, all free induction decays (FIDs) were zero-filled to 240,000 data points and a 0.5 Hz line broadening function was applied. The methyl singlet of the added DSS (set to 0.00 ppm) served both as an internal chemical shift referencing standard and as an internal standard for quantification. All ^1^H-NMR spectra were processed using the Chenomx NMR Suite 8.1 software package (Chenomx Inc., Edmonton, AB, Canada) for compound identification and quantification as previously described [72]. A minimum of three experienced NMR spectroscopists processed and analyzed each NMR spectrum to eliminate compound identification and quantification errors. Sample spike-in experiments were also used to confirm the identity of a number of compounds suspected to be present in specific biofluids or tissue samples. A spike-in experiment involves adding 50–500 μM of the presumptive compound to selected samples to test if the corresponding ^1^H-NMR signals changed as expected. NMR analysis typically led to the identification and quantification of about 50 metabolites in each biofluid or tissue sample.

### 4.7. LC–MS/MS Compound Identification and Quantification 

A targeted, quantitative metabolite profiling approach was employed that combined RPLC–MS with DFI–MS (RPLC–DFI–MS/MS) to determine the concentrations of a wide range of metabolites. These analyses were performed using an in-house quantitative metabolomics assay (TMIC Prime) [73,74]. This assay was used with an Agilent 1260 series ultra high-performance liquid chromatography (UHPLC) system (Agilent Technologies, Palo Alto, CA, USA) coupled with an AB SCIEX QTRAP® 4000 mass spectrometer (Sciex Canada, Concord, ON, Canada) to identify and quantify up to 143 compounds (including amino acids, biogenic amines, glucose, organic acids, acylcarnitines, PCs, LysoPCs, SMs, and SM(OH)s). The absolute quantification of water-soluble compounds including amino acids, organic acids, and biogenic amines was ensured by using two separate UHPLC injections with C18 column separations. On the other hand, glucose and the various lipid classes (acylcarnitines, PCs, LysoPCs, SMs, etc.) are measured by the column-free DFI method (both +ve and –ve mode). While initially designed and calibrated for human metabolomics studies, the measurable ranges of metabolite concentrations available through the TMIC Prime assay match very closely with the known or expected metabolite concentrations in bovine biofluids and tissues (as determined via orthogonal NMR experiments and high levels of agreement with published literature data).

The detection of each metabolite in the TMIC Prime assay relies on multiple reaction monitoring (MRM). The assay incorporates both isotope-labelled internal standards and other quality control (QC) standards into its 96-well filter plate to ensure accurate compound quantification. The first 14 wells in the 96-well plate are used for building calibration curves and QCs, while the other 82 wells are used for sample analysis. For all biofluids analyzed with this assay, both the original sample (without dilution) and diluted samples (10×) were analyzed to ensure correct calibration and quantification. In brief, 10 µL of each sample (the filtered biofluid or the water-soluble or lipid-soluble extract of the tissue) was carefully pipetted into an appropriate sample well of the upper 96-well filter plate and dried using a stream of nitrogen gas. Amino acid and biogenic amine derivatization were done by adding 50 µL of a 5% solution of phenyl-isothiocyanate (PITC) to each well and incubating for 20 min. After incubation and PITC derivatization, the samples were dried down using a nitrogen gas evaporator. The metabolites were then extracted by adding an ammonium acetate/methanol mixture (5 mM ammonium acetate in methanol) to the upper 96-well filter plate, shaking at 330 rpm for 30 min, and then centrifuging the plates so that the extract bled into the lower 96-deep well plate. 

The resulting extract was split for RPLC–MS (150 µL) and DFI–MS (150 µL) analyses followed by a dilution step with 150 µL of water for RPLC–MS analysis and with 400 µL of the MS running solvent for DFI–MS analysis. All LC–MS analyses were conducted on an AB SCIEX QTRAP® 4000 mass spectrometer equipped with an Agilent 1260 series UHPLC system. The Analyst software 1.6.2 (Concord, ON, Canada) was used to control the entire assay’s workflow.

### 4.8. Trace Elemental Analyses Using ICP–MS

All trace elemental analysis was performed on a Perkin-Elmer NexION 350x ICP–MS (Perkin-Elmer, Woodbridge, ON, Canada), operating in a kinetic energy discrimination (KED) mode. Argon (ICP/MS grade, 99.99%) was used as a nebulizer (0.9 mL min^−1^), an auxiliary (1 mL min^−1^) and a plasma gas (15 mL min^−1^). Helium (He) was used as a non-reactive collision gas (Cell gas A: 4.3) to eliminate/minimize chemical interference. The dwell time for each metal ion was set to 50 ms with a total integration time of 500 ms (10 sweeps per reading and three replicates). The uptake of samples/standards/QCs was done by a peristaltic pump using the following protocol: (1) sample flush for 50 s at 48 rpm, (2) read delay for 15 s at 20 rpm, (3) spectral analyses at 20 rpm, and (4) washing for 45 s at 24 rpm. All samples were diluted using 1% HNO_3_, 5% H_2_O_2_, and MiliQ water (grade 1) by a factor of 10 for serum and tissues and a factor of 5 for ruminal fluid samples. Indium (In) was added to the dilution solvent as an internal standard. The final concentration of indium after mixing with the samples/standards was 20 ppb. An external calibration curve was used for the quantitation of all metal ions using a six- to nine-point calibration curve (for each metal) and linear regression. The performance of the ICP–MS was checked daily using a commercially prepared Perkin Elmer Nexion calibration solution to evaluate the sensitivity of the instrument. The Nexion solution was also used to calibrate the mass spectrometer at low (Be), mid (In), and high (U) masses. The accuracy of the ICP–MS analytical protocol was evaluated in every sequence by the analysis of standard reference materials (SRMs)—i.e., serum and water QCs. Continuing calibration verification (CCV) was run every 15 samples to monitor the validity of each calibration curve throughout the sequence.

### 4.9. Literature Research on Bovine Biofluid and Tissue Metabolites 

We conducted an extensive literature review of known bovine biofluids and tissues metabolites and their concentrations using many open-access search engines, such as Google Scholar [75], PubMed [76], and ScienceDirect [77]. We also used several in-house text-mining software packages that were originally developed for the Human Metabolome Project (HMP) and the Human Metabolome Database (HMDB) [78]. Two of the most useful programs were PolySearch [79] and PolySearch2 [80]. These programs are able to take simple keywords (i.e., “serum”, “bovine”, etc.) as input and rapidly create hyperlinked lists of abstracts and papers from PubMed (and other data sources) containing information about bovine metabolites and their corresponding concentration data. PolySearch2 was able to compile a ranked list of bovine metabolites by measuring word co-occurrence frequency using terms such as “cow serum”, “serum”, “beef”, “bovine”, and “cattle” in conjunction with words such as “concentration”, “identification”, “quantification”, “mM”, or “micromol”. PolySearch2 also extracted key sentences from the abstracts, then labelled and hyperlinked the metabolites mentioned in the text. This led to the identification of ~200 papers, abstracts and textbooks with relevant chemical information on bovine biofluids and tissues. 

All literature-derived data regarding bovine compounds, along with their concentrations and references, were compiled, compared, and their names “normalized” to match HMDB [78], chemical abstracts service (CAS) number, and PubChem identifiers. The manually derived compound data was further annotated using an in-house program called DataWrangler [78], which automatically generates names, synonyms, descriptions, structures, chemical taxonomies, physical property data, and bioavailability data. The information generated by DataWrangler was manually checked by three different scientists with post-graduate degrees in biochemistry, physiology, and/or animal sciences. Additional data compiled from two other electronic databases, including the Livestock Metabolome Database (LMDB) [36] and the Milk Composition Database (MCDB) [13] was also collected and cross-checked. After the manual checking phase was complete, the data were then entered into the BMDB. Concentration data were cross-checked manually to identify any large discrepancies (>3×) between entered values. Those that exceeded this threshold were re-analyzed to see if data entry errors had been made. For highly discrepant values, a “majority wins” scheme was used to select the best or most likely value. On the other hand, if our experimental data matched best with one of the discrepant values, that value was selected over other reported value(s). The resulting list of 14 literature-derived bovine biofluids and tissues metabolites (including 278 overlapping experimentally-derived metabolites), along with their concentration data (when available), helped to confirm many of the metabolites and metabolite concentrations previously found in our experimental analyses. 

### 4.10. Genome Scale Inference of Expected Bovine Metabolites

Publicly available bovine metabolite, protein and pathway data from PathBank [39] and UniProt [81] were used to generate a genome-scale compilation of biochemically inferred or “expected” bovine metabolites. These “expected” metabolites correspond to endogenously produced compounds that are very likely to be in cells or tissues, based on well-known or well-characterized biochemical pathways or reactions. Many of these compounds correspond to lipids, transient intermediates, or low-abundance compounds that are not normally measured in metabolomics experiments. Comparisons of these computationally inferred bovine metabolites with metabolite and protein data reported in the HMDB was used to identify potentially missing metabolites or metabolite species. Care was taken to exclude exogenous compounds (human-only food additives, drugs, food constituents, etc.) that would be unlikely to be found in cattle or cattle feed. 

### 4.11. Construction of the BMDB

BMDB was implemented using a Ruby on Rails (http://rubyonrails.org, version 4.2.0) web framework incorporating a MySQL relational database (https://www.mysql.com, version 15.1 Distrib 10.4.6-MariaDB) to manage all of the metabolite data, including descriptions, synonyms, physico-chemical properties, concentrations, spectra, and external references. BMDB was built using HMDB’s framework and is therefore similar in appearance and structure. BMDB uses the model–view–controller architecture, in which internal data logic is separated from user input and data presentation. The raw information stored in the database is dynamically extracted and rendered into web pages. BMDB is hosted on a Digital Ocean server equipped with 4 CPUs, 80 GB of disk space, and 8 GB of RAM.

## 5. Conclusions

Our primary objective for undertaking these studies was to help advance the field of bovine metabolomics. This study used metabolomics techniques, including NMR, LC–MS/MS, and ICP–MS as well as literature reviews facilitated by computer-aided literature mining, to determine the number of experimentally detected or quantified metabolites in different bovine biofluids and tissues. This list of experimentally determined compounds was complemented with computer-aided genome-scale metabolite inference to provide data on thousands of other biochemically “expected” metabolites.

As reported here, the number of metabolites we experimentally detected and measured in serum, ruminal fluid, LT muscle, SM muscle, liver, and testis were 145, 145, 149, 148, 155, and 159, respectively. From this set, a total of 21 compounds or compound species (corresponding to 77 unique structures) are being reported in cattle for the first time, including 3 LysoPCs, 1 SM, 4 PCs, 10 acylcarnitines, and 3 other compounds (NADP, uridine diphosphate glucose, and uridine diphosphate-N-acetylglucosamine), all of which have been added to the BMDB. Our experimentally acquired data corresponds to a total of 3.5% (1834 out of 51,801) of the total number of bovine metabolites/chemicals reported in the BMDB. Our literature-mining efforts identified 1339 (experimentally measured) bovine metabolites, which corresponds to 2.6% of the bovine metabolome. Finally, our genome-scale inference techniques generated 48,628 biochemically expected metabolites, which accounts for the remaining 94% of the bovine metabolome in BMDB.

As far as we are aware, this compilation represents the most complete chemical inventory or chemical assessment of bovine biofluids and tissues that has been achieved to date. All of this information along with other details regarding concentration ranges, chemical structures, names, chemical classes, NMR and MS spectra, other physico-chemical properties and associated references are publicly accessible in the BMDB at www.bovinedb.ca.

Similar to previous metabolomics studies presented from our lab, we wanted to demonstrate the power and potential of quantitative metabolomics to comprehensively characterize common biofluids and tissues in cattle. The results presented here also have implications far beyond the field of metabolomics, especially given the economic importance of the bovine metabolome in the food industry and its importance in human nutrition. We expect these data to serve as a benchmark in comparing various technologies and assessing future methodological improvements in bovine metabolome research. In the meantime, it is hoped that the BMDB will provide a reliable source for metabolomics researchers, animal scientists, food chemists, nutritional scientists, and consumers by providing a comprehensive, easy-to-use and highly centralized web-based resource on the chemical composition of bovine biofluids and tissues. The addition of more samples and the inclusion of more studies will certainly improve the quality and reliability of the data in the BMDB. Indeed, this study is most certainly not the final word on the chemical composition of the bovine metabolome. Over the coming years, we plan to further characterize the bovine biofluids and tissues metabolome using other metabolomics techniques, i.e., GC–MS, GC×GC–MS (to help identify volatile compounds), untargeted high-resolution–mass spectrometry (HR–MS), and more extensive targeted LC–MS/MS techniques to compare and extend the metabolite coverage. 

## Figures and Tables

**Table 1:** List of serum metabolites along with their measured or reported concentrations and their standard deviations and/or ranges (as measured in µM).

Metabolite	Platform	Concentration	Literature Value
**WATER-SOLUBLE COMPOUNDS** ***AMINO ACIDS***			
Alanine *	LC–MS/MS and NMR	240 ± 30	151–222 ^a^
Arginine	LC–MS/MS and NMR	218 ± 33	135–182 ^a^
Asparagine *	LC–MS/MS and NMR	25 ± 4	20–33 ^b^
Aspartate *	LC–MS/MS and NMR	24 ± 11	14–16 ^c^, 31–36 ^a^
Beta-alanine *	NMR	8 ± 1	8–9 ^d^
Citrulline *	LC–MS/MS and NMR	88 ± 15	71–84 ^d^
Creatine	LC–MS/MS and NMR	196 ± 28	
Glutamate *	LC–MS/MS and NMR	92 ± 19	35–39 ^d^, 174–198 ^a^
Glutamine *	LC–MS/MS and NMR	330 ± 42	246–260 ^d^
Glycine *	LC–MS/MS and NMR	398 ± 65	405–428 ^d^
Histidine *	LC–MS/MS	78 ± 10	74–84 ^c^
Isoleucine	LC–MS/MS and NMR	153 ± 14	101–122 ^a^
Leucine *	LC–MS/MS and NMR	212 ± 24	205–264 ^c^
Lysine *	LC–MS/MS and NMR	88 ± 15	58–92 ^b^
Methionine *	LC–MS/MS and NMR	34 ± 4	22–29 ^c^, 46–52 ^a^
Ornithine *	LC–MS/MS and NMR	61 ± 13	62–135 ^a^
Phenylalanine *	LC–MS/MS and NMR	71 ± 7	65–75 ^c^
Proline *	LC–MS/MS and NMR	103 ± 15	84–110 ^a^
Serine *	LC–MS/MS and NMR	85 ± 14	86–89 ^d^
Threonine *	LC–MS/MS and NMR	74 ± 12	58–76 ^c^
Tryptophan *	LC–MS/MS	47 ± 7	37–42 ^c^
Tyrosine	LC–MS/MS and NMR	91 ± 10	68–75 ^c^
Valine *	LC–MS/MS and NMR	356 ± 34	262–322 ^c^
***BIOGENIC AMINES***			
Acetyl-ornithine	LC–MS/MS	3 ± 1	
Asymmetric-dimethylarginine *	LC–MS/MS	1.1 ± 0.2	1.3–2.1 ^e^
Carnosine *	LC–MS/MS	30 ± 12	15–20 ^d^
Creatinine *	LC–MS/MS and NMR	113 ± 17	109–140 ^f^
Kynurenine *	LC–MS/MS	7 ± 2	4–7 ^b^
Methionine-sulfoxide	LC–MS/MS	1.2 ± 0.3	
Methylhistidine *	LC–MS/MS	14 ± 2	2–12 ^g^
Putrescine	LC–MS/MS	0.04 ± 0.02	
Sarcosine	LC–MS/MS and NMR	3 ± 1	10–12 ^d^
Serotonin *	LC–MS/MS	9 ± 3	4–13 ^h^
Spermidine	LC–MS/MS	0.2 ± 0.1	
Spermine	LC–MS/MS	0.2 ± 0.2	
Taurine	LC–MS/MS and NMR	80 ± 20	33–47 ^d^
Total-dimethylarginine	LC–MS/MS	2.1 ± 0.3	
Trans-hydroxyproline	LC–MS/MS	25 ± 5	
Trimethylamine N-oxide	LC–MS/MS	6 ± 3	
***CARBOHYDRATES***			
Glucose *	LC–MS/MS and NMR	3962 ± 443	3290–4070 ^i^
***ORGANIC ACIDS***			
3-hydroxybutyrate *	NMR	340 ± 145	250–2110 ^j^
Acetate	NMR	403 ± 199	920–1040 ^k^
Alpha-aminoadipate	LC–MS/MS	1.28 ± 0.52	7.3–8.1 ^d^
Ascorbate (Vitamin C) *	NMR	11 ± 3	8–18 ^l^
Formate	NMR	78 ± 12	
Fumarate	NMR	1.2 ± 0.2	
Lactate	NMR	4850 ± 2017	658–1600 ^m^
Pyruvate	NMR	150 ± 40	
***MISCELANEOUS***			
Acetone *	NMR	70 ± 22	80–990 ^j^
Betaine	LC–MS/MS and NMR	169 ± 31	14–26 ^n^
Choline	LC–MS/MS and NMR	20 ± 4	4–5 ^n^
Ethanol *	NMR	8 ± 1	3–68 ^i^
Glycerol	NMR	314 ± 38	
Isopropanol	NMR	2 ± 1	
Methanol	NMR	32 ± 4	
Myo-inositol	NMR	45 ± 11	
Urea	NMR	1321 ± 282	1950–4080 ^o^
Uridine	NMR	3 ± 1	
**LIPID-LIKE COMPOUNDS** ***PHOSPHATIDYLCHOLINES, ACYL-ALKYL***			
PC ae (36:0)	LC–MS/MS	1.6 ± 0.4	
PC ae (40:6)	LC–MS/MS	0.46 ± 0.11	
***PHOSPHATIDYLCHOLINES, DIACYL***			
PC aa (32:2)	LC–MS/MS	4 ± 1	
PC aa (36:6)	LC–MS/MS	0.7 ± 0.2	
PC aa (36:0)	LC–MS/MS	6 ± 2	
PC aa (38:6)	LC–MS/MS	1 ± 0.3	
PC aa (38:0)	LC–MS/MS	0.8 ± 0.2	
PC aa (40:6)	LC–MS/MS	1.6 ± 0.4	
PC aa (40:2)	LC–MS/MS	0.4 ± 0.1	
PC aa (40:1)	LC–MS/MS	0.21 ± 0.04	
***LYSOPHOSPHATIDYLCHOLINES, ACYL C***			
LysoPC(14:0)	LC–MS/MS	0.8 ± 0.1	
LysoPC(16:1)	LC–MS/MS	0.6 ± 0.1	
LysoPC(16:0) *	LC–MS/MS	20 ± 4	15–58 ^n^
LysoPC(17:0)	LC–MS/MS	3 ± 1	
LysoPC(18:2)	LC–MS/MS	15 ± 3	30–186 ^n^
LysoPC(18:1)	LC–MS/MS	6 ± 1	18–69 ^n^
LysoPC(18:0) *	LC–MS/MS	30 ± 5	14–82 ^n^
LysoPC(20:4)	LC–MS/MS	0.48 ± 0.14	
LysoPC(20:3)	LC–MS/MS	1.6 ± 0.3	
LysoPC(24:0)	LC–MS/MS	0.051 ± 0.012	
LysoPC(26:1)	LC–MS/MS	0.1 ± 0.04	
LysoPC(26:0)	LC–MS/MS	0.7 ± 0.3	
LysoPC(28:1)	LC–MS/MS	0.3 ± 0.1	
LysoPC(28:0)	LC–MS/MS	0.28 ± 0.11	
***SPHINGOMYELINS***			
SM(16:1)	LC–MS/MS	5 ± 1	
SM(16:0)	LC–MS/MS	68 ± 10	
SM(18:1)	LC–MS/MS	11 ± 3	
SM(18:0)	LC–MS/MS	12 ± 2	
SM(20:2)	LC–MS/MS	1.1 ± 0.3	
***HYDROXYSPHINGOMYELINS***			
SM(14:1(OH))	LC–MS/MS	5 ± 1	
SM(16:1(OH))	LC–MS/MS	9 ± 2	
SM(22:2(OH))	LC–MS/MS	4 ± 1	
SM(22:1(OH))	LC–MS/MS	9 ± 1	
SM(24:1(OH))	LC–MS/MS	2 ± 0.4	
***ACYLCARNITINES***			
C0 (Carnitine)	LC–MS/MS	7 ± 1	
C2 (Acetylcarnitine) *	LC–MS/MS	2 ± 1	0.65–1.09 ^b^
C3:1 (Propenoylcarnitine)	LC–MS/MS	0.029 ± 0.004	
C3 (Propionylcarnitine)	LC–MS/MS	0.2 ± 0.04	
C4:1 (Butenylcarnitine)	LC–MS/MS	0.017 ± 0.002	
C4 (Butyrylcarnitine)	LC–MS/MS	0.2 ± 0.1	
C3-OH (Hydroxypropionylcarnitine) *	LC–MS/MS	0.027 ± 0.004	0.01–0.02 ^b^
C5:1 (Tiglylcarnitine)	LC–MS/MS	0.023 ± 0.004	
C5 (Valerylcarnitine) *	LC–MS/MS	0.09 ± 0.03	0.03–0.06 ^b^
C4-OH (C3-DC) (Hydroxybutyrylcarnitine)	LC–MS/MS	0.04 ± 0.01	
C6:1 (Hexenoylcarnitine)	LC–MS/MS	0.02 ± 0.01	
C6 (C4:1-DC) (Hexanoylcarnitine)	LC–MS/MS	0.05 ± 0.01	0.02–0.03 ^b^
C5-OH (C3-DC-M) (hydroxyvalerylcarnitine) *	LC–MS/MS	0.04 ± 0.01	0.05–0.06 ^b^
C5:1-DC (Glutaconylcarnitine)	LC–MS/MS	0.018 ± 0.003	
C5-DC (C6-OH)(Glutarylcarnitine)	LC–MS/MS	0.03 ± 0.01	
C8 (Octanoylcarnitine)	LC–MS/MS	0.02 ± 0.01	
C5-M-DC (methylglutarylcarnitine)	LC–MS/MS	0.0196 ± 0.0024	
C9 (Nonaylcarnitine)	LC–MS/MS	0.022 ± 0.003	
C7-DC (Pimelylcarnitine) *	LC–MS/MS	0.04 ± 0.04	0.01–0.02 ^b^
C10:2 (Decadienylcarnitine)	LC–MS/MS	0.06 ± 0.01	
C10:1 (Decenoylcarnitine)	LC–MS/MS	0.17 ± 0.03	
C10 (Decanoylcarnitine)	LC–MS/MS	0.18 ± 0.04	
C12:1 (Dodecenoylcarnitine)	LC–MS/MS	0.084 ± 0.014	
C12 (Dodecanoylcarnitine) *	LC–MS/MS	0.04 ± 0.01	0.02–0.03 ^b^
C14:2 (Tetradecadienylcarnitine)	LC–MS/MS	0.03 ± 0.01	
C14:1 (Tetradecenoylcarnitine)	LC–MS/MS	0.0518 ± 0.0103	
C14 (Tetradecanoylcarnitine) *	LC–MS/MS	0.02 ± 0.01	0.01–0.02 ^b^
C12-DC (Dodecanedioylcarnitine)	LC–MS/MS	0.018 ± 0.003	
C14:2-OH (Hydroxytetradecadienylcarnitine)	LC–MS/MS	0.008 ± 0.002	
C14:1-OH (Hydroxytetradecenoylcarnitine)	LC–MS/MS	0.009 ± 0.002	
C16:2 (Hexadecadienylcarnitine)	LC–MS/MS	0.012 ± 0.002	
C16:1 (Hexadecenoylcarnitine)	LC–MS/MS	0.029 ± 0.003	
C16 (Hexadecanoylcarnitine)	LC–MS/MS	0.02 ± 0.01	
C16:2-OH (Hydroxyhexadecadienylcarnitine)	LC–MS/MS	0.005 ± 0.001	
C16:1-OH (Hydroxyhexadecenoylcarnitine)	LC–MS/MS	0.0184 ± 0.0034	
C16-OH (Hydroxyhexadecanoylcarnitine) *	LC–MS/MS	0.008 ± 0.001	0.003–0.006 ^b^
C18:2 (Octadecadienylcarnitine)	LC–MS/MS	0.007 ± 0.001	
C18:1 (Octadecenoylcarnitine)	LC–MS/MS	0.0147 ± 0.0031	
C18 (Octadecanoylcarnitine)	LC–MS/MS	0.021 ± 0.008	
C18:1-OH (Hydroxyoctadecenoylcarnitine) *	LC–MS/MS	0.009 ± 0.001	0.008–0.009 ^b^
**TRACE ELEMENTAL COMPOUNDS**			
Sodium *	ICP–MS	133,515 ± 13,658	107,400–108,600 ^p^, 136,000–136,710 ^q^
Magnesium *	ICP–MS	931 ± 88	850–920 ^f^
Phosphorus *	ICP–MS	1298 ± 164	1350–1620 ^p^
Potassium *	ICP–MS	4296 ± 388	4060–4340 ^f^
Calcium *	ICP–MS	2228 ± 221	1400–2200 ^h^
Iron *	ICP–MS	52 ± 13	50–51 ^r^
Copper *	ICP–MS	9 ± 2	6–9 ^r^
Zinc *	ICP–MS	12 ± 2	14–18 ^r^
Selenium *	ICP–MS	1.4 ± 0.2	0.5–2.7 ^s^
Rubidium	ICP–MS	1.8 ± 0.2	
Strontium	ICP–MS	1 ± 0.1	
Cesium	ICP–MS	0.0017 ± 0.0003	
Barium	ICP–MS	0.2 ± 0.03	

* Compounds that exhibited good agreement with literature values; ^a^ Motyl and Barej, 1986 [40]; ^b^ Sadri et al., 2017 [41]; ^c^ Greenwood et al., 2001 [42]; ^d^ Zhou et al., 2016 [37]; ^e^ Chan et al., 2000 [43]; ^f^ Consolo et al., 2018 [44]; ^g^ van der Drift et al., 2012 [45]; ^h^ Hernandez-Castellano et al., 2017 [46]; ^I^ Raun and Kristensen, 2011 [33]; ^j^ Sato, 2009 [34]; ^k^ Sato et al., 1999 [35]; ^l^ Padilla et al., 2006 [47]; ^m^ Kenny et al., 2002 [48]; ^n^ Artegoitia et al., 2014 [49]; ^o^ Liker et al., 2005 [50]; ^p^ Nozad et al., 2012 [51]; ^q^ Macdonald et al., 2017 [52]; ^r^ Noaman et al., 2012 [30]; ^s^ Waldner et al., 1998 [53].

**Table 2:** Metabolome coverage of different bovine biofluids and tissues in the BMDB.

Tissue/Biofluid Location	Identified Metabolites or Metabolite Species	Identified Metabolites with Unique Structures	Quantified Metabolites with Unique Structures
**BIOFLUID**			
Blood	330	453	296
Colostrum	70	70	4
Milk	928	2350	1652
Ruminal fluid	328	769	728
Semen	76	76	0
Urine	62	62	0
**TISSUE**			
Adipose tissue	199	199	71
Brain	557	1887	0
Epidermis	275	275	0
Fibroblasts	327	327	0
Intestine	253	253	0
Kidney	531	615	0
Liver	1056	1254	273
*Longissimus thoracis* muscle	153	267	267
Mammary gland	269	269	0
Neuron	322	322	0
Pancreas	114	114	0
Placenta	579	586	0
Platelet	204	204	0
Prostate	268	268	0
Semimembranosus muscle	153	267	267
Skeletal muscle	382	496	274
Spleen	168	168	0
Testis	328	442	277
**All tissues**	**857**	**4464**	N/A *

Note: Metabolite species refer to those molecules with non-unique chemical formulas or masses (such as lipid isomers), while metabolites with unique structures correspond to compounds with a unique and clearly defined chemical structure and a unique chemical name; * Not available.

**Table 3:** Comparison of concentrations of 26 metabolites between nuclear magnetic resonance (NMR) and liquid chromatography–tandem mass spectrometry (LC–MS/MS) methods along with their measured or reported concentrations and their standard deviations and/or ranges in bovine serum (as measured in µM).

Compound Name	NMR	LC–MS/MS	Average Difference (%)
Alanine	252 ± 31	240 ± 30	4
Arginine	210 ± 28	218 ± 33	3
Asparagine	26 ± 5	25 ± 4	3
Aspartate	Glucose overlap	24 ± 11	
Betaine	180 ± 37	169 ± 31	6
Choline	19 ± 4	20 ± 4	5
Citrulline	89 ± 16	88 ± 15	1
Creatine	210 ± 30	196 ± 28	6
Creatinine	121 ± 17	113 ± 17	6
Glucose	4572 ± 588	3962 ± 443	14
Glutamate	Proline overlap	92 ± 19	
Glutamine	360 ± 47	330 ± 42	8
Glycine	438 ± 71	398 ± 65	9
Isoleucine	160 ± 22	153 ± 14	4
Leucine	216 ± 30	212 ± 24	1
Lysine	Arginine overlap	88 ± 15	
Methionine	35 ± 5	34 ± 4	2
Ornithine	67 ± 11	61 ± 13	9
Phenylalanine	67 ± 9	71 ± 7	5
Proline	94 ± 16	103 ± 15	9
Sarcosine	4 ± 1	3 ± 1	28
Serine	87 ± 18	85 ± 14	2
Taurine	Glucose overlap	80 ± 20	
Threonine	72 ± 11	74 ± 12	2
Tyrosine	84 ± 11	91 ± 10	8
Valine	390 ± 48	356 ± 34	9

## Supplementary Materials

The following are available online at https://www.mdpi.com/2218-1989/10/6/233/s1, Table S1: List of rumen metabolites along with their measured or reported concentrations and their standard deviations and/or ranges (as measured in µM), Table S2: List of LT and SM muscle metabolites along with their measured concentrations and their standard deviations (as measured in nmol/g), Table S3: List of liver metabolites along with their measured concentrations and their standard deviations (as measured in nmol/g), Table S4: List of testis metabolites along with their measured concentrations and their standard deviations (as measured in nmol/g).
