# Supplementary material for: The Bovine Metabolome
Source: Metabolites. 2020 Jun 5;10(6):233. doi: 10.3390/metabo10060233 (PMC7345087; doi:10.3390/metabo10060233)
Supplement: Supplementary file 1 [file metabolites-10-00233-s001.pdf]

Table S1. List of rumen metabolites along with their measured or reported concentrations and their standard deviations and/or ranges (as measured in μM).

Metabolite	Platform	Concentration	Literature value
**WATER-SOLUBLE COMPOUNDS**			
AMINO ACIDS			
Alanine *	LC–MS/MS & NMR	1060 ± 484	83–799 [a]
Arginine *	LC–MS/MS	17 ± 11	5–81 [b]
Asparagine	LC–MS/MS	1 ± 1	
Aspartate *	LC–MS/MS & NMR	566 ± 204	153–925 [a]
Beta-alanine *	NMR	72 ± 38	3–33 [c]
Citrulline	LC–MS/MS	184 ± 74	
Creatine *	LC–MS/MS & NMR	3 ± 4	2–15 [c]
Glutamate *	LC–MS/MS & NMR	1702 ± 471	238–887 [a]
Glycine *	LC–MS/MS & NMR	974 ± 315	50–696 [a]
Histidine *	LC–MS/MS	44 ± 28	18–69 [a]
Isoleucine *	LC–MS/MS & NMR	556 ± 127	123–1210 [a]
Leucine *	LC–MS/MS & NMR	603 ± 119	76–571 [a]
Lysine *	LC–MS/MS & NMR	2085 ± 869	91–1095 [a]
Methionine	LC–MS/MS & NMR	268 ± 102	9–66 [b]
Ornithine	LC–MS/MS	149 ± 77	7–51 [b]
Phenylalanine	LC–MS/MS & NMR	310 ± 129	17–85 [b]
Proline *	LC–MS/MS & NMR	834 ± 377	240–1275 [a]
Serine	LC–MS/MS & NMR	547 ± 187	24–180 [b]
Threonine	LC–MS/MS & NMR	658 ± 301	53–153 [c]
Tryptophan *	LC–MS/MS & NMR	24 ± 14	4–26 [b]
Tyrosine *	LC–MS/MS & NMR	295 ± 136	68–471 [a]
Valine *	LC–MS/MS & NMR	848 ± 295	71–593 [a]
BIOGENIC AMINES			
Acetyl-ornithine	LC–MS/MS	9 ± 5	0.1–2.5 [b]
Asymmetric-dimethylarginine *	LC–MS/MS	4 ± 3	0.19–1 [b]
Total-dimethylarginine	LC–MS/MS	5 ± 3	19–47 [b]
Histamine *	LC–MS/MS	57 ± 43	1–12 [b]
Methionine-sulfoxide *	LC–MS/MS	26 ± 9	5–37 [b]
Methylamine *	NMR	132 ± 116	27–822 [b]
Methylhistidine	LC–MS/MS	2 ± 1	
Putrescine *	LC–MS/MS & NMR	103 ± 58	16–303 [b]
Sarcosine	LC–MS/MS & NMR	2 ± 1	6–67 [b]
Serotonin	LC–MS/MS	0.1 ± 0.1	0.9–1.1 [b]
Spermidine	LC–MS/MS	37 ± 22	
Spermine	LC–MS/MS	3 ± 3	
Taurine *	LC–MS/MS & NMR	8 ± 7	1–2 [b]
Trans-hydroxyproline	LC–MS/MS	2 ± 1	
CARBOHYDRATES			

Glucose	LC–MS/MS & NMR	15934 ± 11188	393–3111 [b]
Ribose	NMR	886 ± 201	177–501 [b]
ORGANIC ACIDS			
2-oxoglutarate	NMR	40 ± 24	
3-phenylpropionate	NMR	51 ± 18	296–713 [b]
Acetate *	NMR	37264 ± 7582	41000–81000 [a]
Alpha-aminoadipate	LC–MS/MS	1 ± 1	
Butyrate *	NMR	25848 ± 9273	6472–18088 [b]
Formate	NMR	20 ± 4	60–655 [a]
Fumarate *	NMR	18 ± 7	19–315 [a]
Isobutyrate *	NMR	719 ± 226	708–1100 [c]
Lactate *	NMR	2029 ± 1165	224–1560 [a]
Nicotinate *	NMR	60 ± 10	29–79 [b]
Phenylacetate *	NMR	189 ± 57	212–785 [a]
Propionate *	NMR	16103 ± 4550	14000–17000 [a]
Pyroglutamate *	NMR	233 ± 64	311–645 [b]
Pyruvate	NMR	62 ± 33	
Succinate *	NMR	597 ± 344	40–289 [a]
Valerate *	NMR	2739 ± 677	1500–5000 [a]
MISCELANEOUS			
Acetone	NMR	66 ± 40	5–19 [c]
Betaine *	LC–MS/MS	15 ± 40	2–17 [c]
Choline	LC–MS/MS & NMR	105 ± 59	4–40 [c]
Ethanol *	NMR	556 ± 450	600–3200 [d]
Glycerol	NMR	685 ± 231	89–336 [b]
Hypoxanthine	NMR	753 ± 191	38–266 [b]
Isopropanol *	NMR	103 ± 45	14–70 [c]
Thymine	NMR	188 ± 45	15–63 [b]
Uracil	NMR	1007 ± 230	109–405 [c]
Uridine *	NMR	18 ± 10	2–17 [c]
**LIPID-LIKE COMPOUNDS**			
PHOSPHATIDYLCHOLINES, ACYL-ALKYL			
PC ae (36:0)	LC–MS/MS	0.05 ± 0.04	
PC ae (40:6)	LC–MS/MS	0.04 ± 0.01	
PHOSPHATIDYLCHOLINES, DIACYL			
PC aa (32:2)	LC–MS/MS	0.048 ± 0.024	
PC aa (36:0)	LC–MS/MS	0.07 ± 0.04	
PC aa (38:6)	LC–MS/MS	0.06 ± 0.04	
PC aa (38:0)	LC–MS/MS	0.08 ± 0.01	
PC aa (40:6)	LC–MS/MS	0.03 ± 0.01	
PC aa (40:1)	LC–MS/MS	0.016 ± 0.004	
LYSOPHOSPHATIDYLCHOLINES, ACYL C			
LysoPC(14:0)	LC–MS/MS	6 ± 3	
LysoPC(16:0) *	LC–MS/MS	0.3 ± 0.2	0.01–0.2 [b]
LysoPC(18:2)	LC–MS/MS	0.2 ± 0.1	

LysoPC(18:1)	LC–MS/MS	0.074 ± 0.051	
LysoPC(18:0)	LC–MS/MS	0.2 ± 0.1	
LysoPC(20:4)	LC–MS/MS	0.03 ± 0.02	
LysoPC(20:3)	LC–MS/MS	2 ± 1	
LysoPC(24:0)	LC–MS/MS	0.161 ± 0.153	
LysoPC(26:0)	LC–MS/MS	0.7 ± 0.3	
LysoPC(28:0)	LC–MS/MS	0.11 ± 0.03	
SPHINGOMYELINS			
SM(16:0)	LC–MS/MS	0.75 ± 0.62	0.01–0.05 [b]
SM(18:1)	LC–MS/MS	0.04 ± 0.03	
SM(18:0)	LC–MS/MS	0.19 ± 0.13	
HYDROXYSPHINGOMYELINS			
SM(14:1(OH))	LC–MS/MS	0.05 ± 0.01	0.01–0.03 [b]
SM(16:1(OH))	LC–MS/MS	0.07 ± 0.04	0.01–0.02 [b]
SM(22:1(OH)) *	LC–MS/MS	0.1 ± 0.1	0.01–0.1 [b]
ACYLCARNITINES			
C0 (Carnitine)	LC–MS/MS	3 ± 1	
C2 (Acetylcarnitine)	LC–MS/MS	0.24 ± 0.11	
C3:1 (Propenoylcarnitine)	LC–MS/MS	0.03 ± 0.01	
C3 (Propionylcarnitine)	LC–MS/MS	0.09 ± 0.02	0.03–0.05 [b]
C4:1 (Butenylcarnitine) *	LC–MS/MS	0.04 ± 0.01	0.03–0.04 [b]
C4 (Butyrylcarnitine)	LC–MS/MS	0.05 ± 0.02	
C3-OH (Hydroxypropionylcarnitine) *	LC–MS/MS	0.04 ± 0.01	0.03–0.04 [b]
C5:1 (Tiglylcarnitine) *	LC–MS/MS	0.03 ± 0.01	0.04–0.07 [b]
C5 (Valerylcarnitine) *	LC–MS/MS	0.03 ± 0.01	0.03–0.06 [b]
C4-OH (C3-DC) (Hydroxybutyrylcarnitine)	LC–MS/MS	1 ± 1	0.04–0.33 [b]
C6:1 (Hexenoylcarnitine)	LC–MS/MS	0.04 ± 0.01	0.09–0.10 [b]
C6 (C4:1-DC) (Hexanoylcarnitine)	LC–MS/MS	0.08 ± 0.01	
C5-OH (C3-DC-M) (hydroxyvalerylcarnitine)	LC–MS/MS	0.027 ± 0.004	
C5:1-DC (Glutaconylcarnitine)	LC–MS/MS	0.019 ± 0.003	0.039–0.045 [b]
C5-DC (C6-OH)(Glutarylcarnitine)	LC–MS/MS	0.015 ± 0.003	
C8 (Octanoylcarnitine)	LC–MS/MS	0.014 ± 0.003	
C5-M-DC (methylglutarylcarnitine)	LC–MS/MS	0.022 ± 0.003	0.174–0.2 [b]
C9 (Nonaylcarnitine)	LC–MS/MS	0.011 ± 0.002	
C7-DC (Pimelylcarnitine) *	LC–MS/MS	0.029 ± 0.021	0.03–0.07 [b]
C10:2 (Decadienylcarnitine)	LC–MS/MS	0.06 ± 0.01	
C10:1 (Decenoylcarnitine)	LC–MS/MS	0.2 ± 0.1	
C10 (Decanoylcarnitine)	LC–MS/MS	0.1 ± 0.02	
C12:1 (Dodecenoylcarnitine)	LC–MS/MS	0.1 ± 0.02	
C12 (Dodecanoylcarnitine)	LC–MS/MS	0.03 ± 0.01	
C14:2 (Tetradecadienylcarnitine) *	LC–MS/MS	0.023 ± 0.004	0.017–0.023 [b]
C14:1 (Tetradecenoylcarnitine)	LC–MS/MS	0.007 ± 0.002	0.089–0.091 [b]
C14 (Tetradecanoylcarnitine)	LC–MS/MS	0.009 ± 0.002	
C12-DC (Dodecanedioylcarnitine)	LC–MS/MS	0.037 ± 0.012	
C14:2-OH (Hydroxytetradecadienylcarnitine) *	LC–MS/MS	0.011 ± 0.003	0.008–0.032 [b]

C14:1-OH (Hydroxytetradecenoylcarnitine)	LC–MS/MS	0.009 ± 0.002	
C16:2 (Hexadecadienylcarnitine) *	LC–MS/MS	0.008 ± 0.001	0.002–0.013 [b]
C16:1 (Hexadecenoylcarnitine)	LC–MS/MS	0.023 ± 0.004	
C16 (Hexadecanoylcarnitine) *	LC–MS/MS	0.016 ± 0.004	0.01–0.055 [b]
C16:2-OH (Hydroxyhexadecadienylcarnitine)	LC–MS/MS	0.009 ± 0.002	
C16:1-OH (Hydroxyhexadecenoylcarnitine)	LC–MS/MS	0.01 ± 0.002	
C16-OH (Hydroxyhexadecanoylcarnitine) *	LC–MS/MS	0.012 ± 0.003	0.009–0.011 [b]
C18:2 (Octadecadienylcarnitine)	LC–MS/MS	0.006 ± 0.001	0.07–0.072 [b]
C18:1 (Octadecenoylcarnitine)	LC–MS/MS	0.015 ± 0.003	
C18 (Octadecanoylcarnitine)	LC–MS/MS	0.0067 ± 0.0021	
C18:1-OH (Hydroxyoctadecenoylcarnitine) *	LC–MS/MS	0.012 ± 0.003	0.002–0.022 [b]
**TRACE ELEMENTAL COMPOUNDS**			
Lithium	ICP–MS	21 ± 5	3–4 [b]
Sodium	ICP–MS	235634 ± 19788	110000–117000 [b]
Magnesium	ICP–MS	7465 ± 3511	96–108 [b]
Phosphorus	ICP–MS	12395 ± 1908	9140–9270 [b]
Potassium	ICP–MS	39878 ± 6579	17980–18270 [b]
Calcium *	ICP–MS	371 ± 722	904–958 [b]
Titanium	ICP–MS	2.5 ± 0.4	
Manganese *	ICP–MS	59 ± 62	2–3 [b]
Iron *	ICP–MS	40 ± 8	21–32 [b]
Nickel	ICP–MS	2 ± 1	0.04–0.2 [b]
Cobalt	ICP–MS	1 ± 0.2	
Copper	ICP–MS	5 ± 1	2–3 [b]
Zinc	ICP–MS	10 ± 3	2–3 [b]
Rubidium	ICP–MS	25 ± 5	5–6 [b]
Strontium *	ICP–MS	4 ± 2	1–2 [b]
Cesium	ICP–MS	0.03 ± 0.01	0.001–0.01 [b]
Barium *	ICP–MS	2 ± 1	0.5–1 [b]

* Compounds that exhibited good agreement with literature values; [a] Lee et al., 2012 [31]; [b] Saleem et al., 2013 [12]; [c] O'Callaghan et al., 2018 [29]; [d] Raun and Kristensen, 2011 [33].

Table S2. List of LT and SM muscle metabolites along with their measured concentrations and their standard deviations (as measured in nmol/g).

Metabolite	Platform	Concentration (LT muscle)	Concentration (SM muscle)	Literature value
<u>**WATER-SOLUBLE COMPOUNDS**</u>				
AMINO ACIDS				
Alanine *	LC–MS/MS & NMR	1465 ± 272	1472 ± 278	1055–1310 [a]
Arginine	LC–MS/MS	61 ± 14	70 ± 16	299–686 [b]
Asparagine	LC–MS/MS	40 ± 12	46 ± 17	106–225 [b]
Aspartate *	LC–MS/MS & NMR	70 ± 28	57 ± 28	14–54 [b]
Beta-alanine *	NMR	149 ± 57	122 ± 37	84–155 [b]
Citrulline	LC–MS/MS	34 ± 12	31 ± 14	59–111 [b]
Creatine *	LC–MS/MS & NMR	4672 ± 438	4755 ± 306	1040–1520 [c], 7262–10319 [a]
Glutamate *	LC–MS/MS & NMR	425 ± 180	541 ± 234	134–942 [b]
Glutamine *	LC–MS/MS & NMR	2841 ± 828	2492 ± 668	3051–4834 [b]
Glutathione	NMR	226 ± 51	162 ± 46	769–775 [b]
Glycine *	LC–MS/MS & NMR	576 ± 215	538 ± 147	342–570 [a]
Histidine	LC–MS/MS	75 ± 18	85 ± 20	134–281 [b]
Isoleucine *	LC–MS/MS & NMR	107 ± 22	116 ± 23	43–231 [a]
Leucine *	LC–MS/MS & NMR	154 ± 37	158 ± 33	80–424 [a]
Lysine	LC–MS/MS	59 ± 18	66 ± 19	306–745 [b]
Methionine *	LC–MS/MS & NMR	34 ± 10	37 ± 11	40–395 [b]
Ornithine	LC–MS/MS	35 ± 12	40 ± 10	80–187 [b]
Phenylalanine *	LC–MS/MS & NMR	55 ± 12	58 ± 10	51–258 [a]
Proline	LC–MS/MS	174 ± 59	203 ± 60	240–365 [b]
Serine	LC–MS/MS	165 ± 36	161 ± 31	273–830 [b]
Threonine *	LC–MS/MS & NMR	194 ± 80	193 ± 32	193–468 [b]
Tryptophan	LC–MS/MS & NMR	19 ± 5	21 ± 3	35–95 [b]
Tyrosine *	LC–MS/MS & NMR	54 ± 14	52 ± 10	44–269 [a]
Valine *	LC–MS/MS & NMR	270 ± 62	278 ± 49	71–288 [a]
BIOGENIC AMINES				
Acetyl-ornithine	LC–MS/MS	18 ± 8	21 ± 8	
Asymmetric-dimethylarginine	LC–MS/MS	0.8 ± 0.2	0.9 ± 0.2	
Carnosine	LC–MS/MS & NMR	22085 ± 4859	21958 ± 3048	9690–13658 [b]
Creatinine *	LC–MS/MS & NMR	313 ± 48	315 ± 47	128–429 [a]
Histamine	LC–MS/MS	17 ± 4	23 ± 6	
Kynurenine	LC–MS/MS	0.6 ± 0.2	0.5 ± 0.2	
Methionine-sulfoxide	LC–MS/MS	3 ± 7	2 ± 8	
Methylhistidine	LC–MS/MS	35 ± 8	35 ± 9	
Putrescine	LC–MS/MS	1.3 ± 0.4	2 ± 1	9–22 [b]
Sarcosine	LC–MS/MS & NMR	12 ± 5	10 ± 4	
Spermidine	LC–MS/MS	0.09 ± 0.03	0.11 ± 0.04	
Spermine	LC–MS/MS	0.2 ± 0.1	0.11 ± 0.03	
Taurine	LC–MS/MS & NMR	480 ± 146	844 ± 301	

Total-dimethylarginine	LC–MS/MS	1.7 ± 0.4	2 ± 1	
Trans-hydroxyproline	LC–MS/MS	32 ± 11	31 ± 11	
Trimethylamine N-oxide	LC–MS/MS	14 ± 5	14 ± 4	
CARBOHYDRATES				
Glucose-1-phosphate *	NMR	184 ± 58	175 ± 48	120 [d], 363–1178 [b]
Glucose	LC–MS/MS & NMR	536 ± 259	754 ± 403	3300 [d]
ORGANIC ACIDS				
3-hydroxybutyrate *	NMR	97 ± 27	88 ± 33	114–242 [b]
Acetate *	NMR	188 ± 31	219 ± 52	97–291 [a]
Alpha-aminoadipate	LC–MS/MS	7 ± 2	10 ± 4	
Formate	NMR	533 ± 316	723 ± 184	110 [d]
Fumarate *	NMR	141 ± 64	220 ± 76	36–208 [b]
Inosinate (IMP) *	NMR	15 ± 11	124 ± 136	31–59 [c], 40–8977 [b]
Lactate *	NMR	31131 ± 8268	37879 ± 10580	14262–21850 [a], 37175–131553 [b]
Nicotinurate	NMR	96 ± 38	170 ± 54	
Pyruvate *	NMR	123 ± 51	157 ± 68	1–183 [b]
Succinate *	NMR	1133 ± 308	743 ± 384	891–2314 [b]
MISCELANEOUS				
Betaine *	LC–MS/MS & NMR	1321 ± 351	1139 ± 246	1477–1826 [b]
Choline	LC–MS/MS & NMR	15 ± 7	15 ± 6	34–153 [b]
Ethanol *	NMR	299 ± 182	246 ± 172	160 [d]
Glycerol *	NMR	328 ± 210	326 ± 174	392–528 [c]
Myo-inositol	NMR	504 ± 133	593 ± 126	
NAD+ *	NMR	641 ± 127	515 ± 70	15–611 [b]
NADH	NMR	94 ± 75	101 ± 45	
O-acetylcarnitine	NMR	1892 ± 398	1612 ± 318	
**LIPID-LIKE COMPOUNDS**				
PHOSPHATIDYLCHOLINES, ACYL-ALKYL				
PC ae (36:0)	LC–MS/MS	0.55 ± 0.12	0.6 ± 0.2	
PC ae (40:6)	LC–MS/MS	0.44 ± 0.12	0.56 ± 0.14	
PHOSPHATIDYLCHOLINES, DIACYL				
PC aa (32:2)	LC–MS/MS	1.7 ± 0.4	3 ± 2	
PC aa (36:6)	LC–MS/MS	0.3 ± 0.1	0.3 ± 0.1	
PC aa (36:0)	LC–MS/MS	0.85 ± 0.14	1 ± 0.2	
PC aa (38:6)	LC–MS/MS	0.5 ± 0.1	0.6 ± 0.2	
PC aa (38:0)	LC–MS/MS	0.4 ± 0.1	0.4 ± 0.1	
PC aa (40:6)	LC–MS/MS	0.13 ± 0.03	0.14 ± 0.04	
PC aa (40:2)	LC–MS/MS	0.017 ± 0.004	0.02 ± 0.01	
PC aa (40:1)	LC–MS/MS	0.026 ± 4	0.031 ± 0.004	
LYSOPHOSPHATIDYLCHOLINES, ACYL C				
LysoPC(14:0)	LC–MS/MS	0.07 ± 0.01	0.07 ± 0.01	
LysoPC(16:1)	LC–MS/MS	0.02 ± 0.01	0.03 ± 0.01	
LysoPC(16:0)	LC–MS/MS	0.1 ± 0.02	0.11 ± 0.03	

LysoPC(17:0)	LC–MS/MS	0.013 ± 0.003	0.016 ± 0.004	
LysoPC(18:2)	LC–MS/MS	0.3 ± 0.1	0.4 ± 0.1	
LysoPC(18:1)	LC–MS/MS	0.3 ± 0.1	0.4 ± 0.1	
LysoPC(18:0)	LC–MS/MS	0.07 ± 0.02	0.08 ± 0.03	
LysoPC(20:4)	LC–MS/MS	0.07 ± 0.03	0.09 ± 0.03	
LysoPC(20:3)	LC–MS/MS	0.05 ± 0.01	0.08 ± 0.02	
LysoPC(24:0)	LC–MS/MS	0.023 ± 0.004	0.03 ± 0.01	
LysoPC(26:1)	LC–MS/MS	0.05 ± 0.01	0.06 ± 0.02	
LysoPC(26:0)	LC–MS/MS	0.05 ± 0.01	0.06 ± 0.02	
LysoPC(28:1)	LC–MS/MS	0.04 ± 0.01	0.04 ± 0.01	
LysoPC(28:0)	LC–MS/MS	0.05 ± 0.01	0.06 ± 0.02	
SPHINGOMYELINS				
SM(16:1)	LC–MS/MS	0.11 ± 0.02	0.13 ± 0.03	
SM(16:0)	LC–MS/MS	3 ± 1	2.45 ± 0.71	
SM(18:1)	LC–MS/MS	1.1 ± 0.2	1.2 ± 0.4	
SM(18:0)	LC–MS/MS	8 ± 1	8 ± 2	
SM(20:2)	LC–MS/MS	0.4 ± 0.1	0.5 ± 0.2	
HYDROXYSPHINGOMYELINS				
SM(14:1(OH))	LC–MS/MS	0.2 ± 0.04	0.2 ± 0.1	
SM(16:1(OH))	LC–MS/MS	1.1 ± 0.2	1.2 ± 0.4	
SM(22:2(OH))	LC–MS/MS	0.4 ± 0.1	0.47 ± 0.1	
SM(22:1(OH))	LC–MS/MS	0.9 ± 0.2	0.94 ± 0.22	
SM(24:1(OH))	LC–MS/MS	0.08 ± 0.02	0.09 ± 0.03	
ACYLCARNITINES				
C0 (Carnitine) *	LC–MS/MS	1856 ± 424	1751 ± 337	791–1143 [a], 2600 [d]
C2 (Acetylcarnitine)	LC–MS/MS	854 ± 153	871 ± 150	
C3:1 (Propenoylcarnitine)	LC–MS/MS	1.8 ± 0.2	1.8 ± 0.2	
C3 (Propionylcarnitine)	LC–MS/MS	6 ± 2	7 ± 2	
C4:1 (Butenylcarnitine)	LC–MS/MS	0.07 ± 0.01	0.08 ± 0.03	
C4 (Butyrylcarnitine)	LC–MS/MS	16 ± 6	22 ± 8	
C3-OH (Hydroxypropionylcarnitine)	LC–MS/MS	0.2 ± 0.1	0.2 ± 0.1	
C5:1 (Tiglylcarnitine)	LC–MS/MS	0.3 ± 0.1	0.3 ± 0.1	
C5 (Valerylcarnitine)	LC–MS/MS	4 ± 2	4 ± 2	
C4-OH (C3-DC) (Hydroxybutyrylcarnitine)	LC–MS/MS	6 ± 3	7 ± 5	
C6:1 (Hexenoylcarnitine)	LC–MS/MS	0.12 ± 0.03	0.2 ± 0.1	
C6 (C4:1-DC) (Hexanoylcarnitine)	LC–MS/MS	0.52 ± 0.51	0.7 ± 0.4	
C5-OH (C3-DC-M) (hydroxyvalerylcarnitine)	LC–MS/MS	1.6 ± 0.4	1.7 ± 0.4	
C5:1-DC (Glutaconylcarnitine)	LC–MS/MS	0.04 ± 0.03	0.05 ± 0.03	
C5-DC (C6-OH)(Glutarylcarnitine)	LC–MS/MS	0.09 ± 0.04	0.14 ± 0.11	
C8 (Octanoylcarnitine)	LC–MS/MS	0.26 ± 0.32	0.27 ± 0.24	
C5-M-DC (methylglutarylcarnitine)	LC–MS/MS	0.02 ± 0.01	0.02 ± 0.01	
C9 (Nonaylcarnitine)	LC–MS/MS	0.03 ± 0.02	0.04 ± 0.03	
C7-DC (Pimelylcarnitine)	LC–MS/MS	0.1 ± 0.1	0.14 ± 0.11	
C10:2 (Decadienylcarnitine)	LC–MS/MS	0.04 ± 0.01	0.024 ± 0.004	

C10:1 (Decenoylcarnitine)	LC–MS/MS	0.3 ± 0.1	0.3 ± 0.1
C10 (Decanoylcarnitine)	LC–MS/MS	0.2 ± 0.2	0.2 ± 0.2
C12:1 (Dodecenoylcarnitine)	LC–MS/MS	0.12 ± 0.03	0.11 ± 0.02
C12 (Dodecanoylcarnitine)	LC–MS/MS	0.1 ± 0.1	0.04 ± 0.03
C14:2 (Tetradecadienylcarnitine)	LC–MS/MS	0.011 ± 0.002	0.009 ± 0.002
C14:1 (Tetradecenoylcarnitine)	LC–MS/MS	0.01 ± 0.01	0.01 ± 0.01
C14 (Tetradecanoylcarnitine)	LC–MS/MS	0.02 ± 0.01	0.01 ± 0.01
C12-DC (Dodecanedioylcarnitine)	LC–MS/MS	0.017 ± 0.004	0.016 ± 0.002
C14:2-OH (Hydroxytetradecadienylcarnitine)	LC–MS/MS	0.006 ± 0.001	0.005 ± 0.001
C14:1-OH (Hydroxytetradecenoylcarnitine)	LC–MS/MS	0.008 ± 0.004	0.009 ± 0.004
C16:2 (Hexadecadienylcarnitine)	LC–MS/MS	0.007 ± 0.001	0.007 ± 0.001
C16:1 (Hexadecenoylcarnitine)	LC–MS/MS	0.032 ± 0.004	0.032 ± 0.004
C16 (Hexadecanoylcarnitine)	LC–MS/MS	0.02 ± 0.01	0.009 ± 0.004
C16:2-OH (Hydroxyhexadecadienylcarnitine)	LC–MS/MS	0.006 ± 0.001	0.007 ± 0.001
C16:1-OH (Hydroxyhexadecenoylcarnitine)	LC–MS/MS	0.011 ± 0.003	0.011 ± 0.004
C16-OH (Hydroxyhexadecanoylcarnitine)	LC–MS/MS	0.008 ± 0.002	0.007 ± 0.003
C18:2 (Octadecadienylcarnitine)	LC–MS/MS	0.004 ± 0.001	0.004 ± 0.001
C18:1 (Octadecenoylcarnitine)	LC–MS/MS	0.008 ± 0.004	0.007 ± 0.003
C18 (Octadecanoylcarnitine)	LC–MS/MS	0.02 ± 0.01	0.006 ± 0.002
C18:1-OH (Hydroxyoctadecenoylcarnitine)	LC–MS/MS	0.009 ± 0.002	0.01 ± 0.01
**TRACE ELEMENTAL COMPOUNDS**			
Lithium	ICP–MS	1.5 ± 0.4	1.5 ± 0.3
Boron	ICP–MS	<LOQ[1]	36 ± 18
Sodium	ICP–MS	10109 ± 2109	12037 ± 2852
Magnesium	ICP–MS	279 ± 84	286 ± 105
Phosphorus	ICP–MS	21915 ± 3094	21818 ± 2594
Potassium	ICP–MS	49300 ± 7605	51519 ± 7094
Titanium	ICP–MS	1.2 ± 0.2	1.3 ± 0.2
Vanadium	ICP–MS	0.013 ± 0.004	0.009 ± 0.001
Manganese	ICP–MS	0.12 ± 0.02	<LOQ
Iron	ICP–MS	10 ± 4	8 ± 3
Cobalt	ICP–MS	0.02 ± 0.01	0.022 ± 0.003
Copper	ICP–MS	0.25 ± 0.13	0.2 ± 0.1
Rubidium	ICP–MS	37 ± 7	40 ± 7
Strontium	ICP–MS	0.06 ± 0.01	<LOQ
Cesium	ICP–MS	0.03 ± 0.01	0.03 ± 0.01
Thallium	ICP–MS	0.0007 ± 0.0002	0.0009 ± 0.0001

[*] Compounds that exhibited good agreement with literature values; [1] LOQ: limit of quantification; [a] Jung et al., 2010 [7]; [b] Muroya et al., 2019 [8]; [c] Kodani et al., 2017 [58]; [d] Kim et al., 2016 [57].

Table S3. List of liver metabolites along with their measured concentrations and their standard deviations (as measured in nmol/g).

Metabolite	Platform	Concentration	Literature value
**WATER-SOLUBLE COMPOUNDS**			
AMINO ACIDS			
Alanine	LC–MS/MS & NMR	1388 ± 276	2366–3515 [a]
Arginine	LC–MS/MS	9 ± 5	
Asparagine	LC–MS/MS	169 ± 37	
Aspartate	LC–MS/MS & NMR	384 ± 165	
Beta-alanine	NMR	750 ± 137	
Citrulline	LC–MS/MS	20 ± 5	
Creatine	LC–MS/MS & NMR	1321 ± 464	
Glutamate	LC–MS/MS & NMR	4092 ± 928	6122–7999 [a]
Glutamine	LC–MS/MS & NMR	1433 ± 231	1911–2576 [a]
Glutathione	LC–MS/MS & NMR	892 ± 472	
Glycine	NMR	3201 ± 499	
Histidine	LC–MS/MS	382 ± 67	
Isoleucine	LC–MS/MS & NMR	178 ± 29	
Leucine	LC–MS/MS & NMR	426 ± 117	
Lysine	LC–MS/MS	115 ± 33	
Methionine	LC–MS/MS & NMR	54 ± 16	
Ornithine	LC–MS/MS & NMR	270 ± 63	984–1184 [a]
Phenylalanine	LC–MS/MS & NMR	138 ± 22	
Proline	LC–MS/MS	352 ± 67	
Serine	LC–MS/MS	422 ± 114	
Threonine	LC–MS/MS & NMR	377 ± 95	
Tryptophan	LC–MS/MS & NMR	44 ± 7	
Tyrosine	LC–MS/MS & NMR	84 ± 27	
Valine	LC–MS/MS & NMR	408 ± 73	
BIOGENIC AMINES			
Acetyl-ornithine	LC–MS/MS	23 ± 8	
Asymmetric-dimethylarginine	LC–MS/MS	1 ± 1	
Carnosine	LC–MS/MS & NMR	393 ± 130	
Creatinine	LC–MS/MS & NMR	58 ± 12	
Dopamine	LC–MS/MS	2 ± 2	
Histamine	LC–MS/MS	25 ± 16	
Kynurenine	LC–MS/MS	3 ± 2	
Methionine-sulfoxide	LC–MS/MS	13 ± 28	
Methylhistidine	LC–MS/MS	25 ± 5	
Putrescine	LC–MS/MS	0.2 ± 0.1	
Sarcosine	LC–MS/MS & NMR	23 ± 10	
Serotonin	LC–MS/MS	2 ± 1	
Taurine	LC–MS/MS & NMR	2243 ± 1186	
Total-dimethylarginine	LC–MS/MS	4 ± 1	

Trans-hydroxyproline	LC–MS/MS	51 ± 13
CARBOHYDRATES		
Glucose	LC–MS/MS & NMR	80098 ± 14629
ORGANIC ACIDS		
3-hydroxybutyrate	NMR	355 ± 89
Acetate	NMR	201 ± 91
Alpha-aminoadipate	LC–MS/MS	68 ± 27
Ascorbate (Vitamin C)	NMR	880 ± 257
Formate	NMR	1686 ± 209
Fumarate	NMR	299 ± 55
Inosinate (IMP)	NMR	34 ± 27
Lactate	NMR	12311 ± 1719
Nicotinurate	NMR	641 ± 87
Pyruvate	NMR	108 ± 20
Succinate	NMR	1194 ± 584
MISCELANEOUS		
Betaine	LC–MS/MS & NMR	358 ± 93
Choline	LC–MS/MS & NMR	345 ± 109
Ethanol	NMR	2065 ± 985
Glycerol	NMR	6219 ± 1151
Hypoxanthine	NMR	1227 ± 156
Inosine	NMR	833 ± 140
Myo-inositol	NMR	1177 ± 221
NAD+	NMR	347 ± 99
NADH	NMR	48 ± 26
NADP+	NMR	16 ± 8
O-phosphocholine	NMR	1658 ± 593
sn-Glycero-3-phosphocholine	NMR	10833 ± 1521
Uridine monophosphate (UMP)	NMR	58 ± 31
Uracil	NMR	76 ± 30
Uridine	NMR	344 ± 86
**LIPID-LIKE COMPOUNDS**		
PHOSPHATIDYLCHOLINES, ACYL-ALKYL		
PC ae (36:0)	LC–MS/MS	5 ± 2
PC ae (40:6)	LC–MS/MS	2 ± 1
PHOSPHATIDYLCHOLINES, DIACYL		
PC aa (32:2)	LC–MS/MS	3 ± 1
PC aa (36:6)	LC–MS/MS	3 ± 1
PC aa (36:0)	LC–MS/MS	17 ± 5
PC aa (38:6)	LC–MS/MS	6 ± 3
PC aa (38:0)	LC–MS/MS	1.6 ± 0.4
PC aa (40:6)	LC–MS/MS	14 ± 6
PC aa (40:2)	LC–MS/MS	0.7 ± 0.2
PC aa (40:1)	LC–MS/MS	0.6 ± 0.2
LYSOPHOSPHATIDYLCHOLINES, ACYL C		

LysoPC(14:0)	LC–MS/MS	0.09 ± 0.01
LysoPC(16:1)	LC–MS/MS	0.06 ± 0.02
LysoPC(16:0)	LC–MS/MS	0.7 ± 0.2
LysoPC(17:0)	LC–MS/MS	0.15 ± 0.03
LysoPC(18:2)	LC–MS/MS	1.1 ± 0.3
LysoPC(18:1)	LC–MS/MS	0.87 ± 0.23
LysoPC(18:0)	LC–MS/MS	2.1 ± 0.4
LysoPC(20:4)	LC–MS/MS	0.41 ± 0.11
LysoPC(20:3)	LC–MS/MS	0.132 ± 0.051
LysoPC(24:0)	LC–MS/MS	0.06 ± 0.01
LysoPC(26:1)	LC–MS/MS	0.04 ± 0.01
LysoPC(26:0)	LC–MS/MS	0.09 ± 0.03
LysoPC(28:1)	LC–MS/MS	0.1 ± 0.02
LysoPC(28:0)	LC–MS/MS	0.2 ± 0.1
SPHINGOMYELINS		
SM(16:1)	LC–MS/MS	0.9 ± 0.3
SM(16:0)	LC–MS/MS	22 ± 7
SM(18:1)	LC–MS/MS	3 ± 1
SM(18:0)	LC–MS/MS	6 ± 2
SM(20:2)	LC–MS/MS	0.8 ± 0.3
HYDROXYSPHINGOMYELINS		
SM(14:1(OH))	LC–MS/MS	2 ± 1
SM(16:1(OH))	LC–MS/MS	5 ± 1
SM(22:2(OH))	LC–MS/MS	4 ± 1
SM(22:1(OH))	LC–MS/MS	14 ± 3
SM(24:1(OH))	LC–MS/MS	4 ± 1
ACYLCARNITINES		
C0 (Carnitine)	LC–MS/MS	22 ± 6
C2 (Acetylcarnitine)	LC–MS/MS	5 ± 2
C3:1 (Propenoylcarnitine)	LC–MS/MS	0.2 ± 0.1
C3 (Propionylcarnitine)	LC–MS/MS	3 ± 1
C4:1 (Butenylcarnitine)	LC–MS/MS	0.08 ± 0.01
C4 (Butyrylcarnitine)	LC–MS/MS	0.36 ± 0.08
C3-OH (Hydroxypropionylcarnitine)	LC–MS/MS	0.07 ± 0.02
C5:1 (Tiglylcarnitine)	LC–MS/MS	0.022 ± 0.004
C5 (Valerylcarnitine)	LC–MS/MS	0.17 ± 0.04
C4-OH (C3-DC) (Hydroxybutyrylcarnitine)	LC–MS/MS	0.09 ± 0.02
C6:1 (Hexenoylcarnitine)	LC–MS/MS	0.17 ± 0.03
C6 (C4:1-DC) (Hexanoylcarnitine)	LC–MS/MS	0.3 ± 0.1
C5-OH (C3-DC-M) (hydroxyvalerylcarnitine)	LC–MS/MS	0.09 ± 0.02
C5:1-DC (Glutaconylcarnitine)	LC–MS/MS	0.026 ± 0.004
C5-DC (C6-OH)(Glutarylcarnitine)	LC–MS/MS	0.2 ± 0.1
C8 (Octanoylcarnitine)	LC–MS/MS	0.09 ± 0.02
C5-M-DC (methylglutarylcarnitine)	LC–MS/MS	0.11 ± 0.02
C9 (Nonaylcarnitine)	LC–MS/MS	0.04 ± 0.01

C7-DC (Pimelylcarnitine)	LC–MS/MS	0.14 ± 0.11
C10:2 (Decadienylcarnitine)	LC–MS/MS	0.04 ± 0.01
C10:1 (Decenoylcarnitine)	LC–MS/MS	0.3 ± 0.1
C10 (Decanoylcarnitine)	LC–MS/MS	0.16 ± 0.03
C12:1 (Dodecenoylcarnitine)	LC–MS/MS	0.3 ± 0.1
C12 (Dodecanoylcarnitine)	LC–MS/MS	0.031 ± 0.004
C14:2 (Tetradecadienylcarnitine)	LC–MS/MS	0.011 ± 0.002
C14:1 (Tetradecenoylcarnitine)	LC–MS/MS	0.011 ± 0.002
C14 (Tetradecanoylcarnitine)	LC–MS/MS	0.011 ± 0.003
C12-DC (Dodecanedioylcarnitine)	LC–MS/MS	0.021 ± 0.003
C14:2-OH (Hydroxytetradecadienylcarnitine)	LC–MS/MS	0.008 ± 0.001
C14:1-OH (Hydroxytetradecenoylcarnitine)	LC–MS/MS	0.009 ± 0.002
C16:2 (Hexadecadienylcarnitine)	LC–MS/MS	0.008 ± 0.001
C16:1 (Hexadecenoylcarnitine)	LC–MS/MS	0.032 ± 0.003
C16 (Hexadecanoylcarnitine)	LC–MS/MS	0.012 ± 0.004
C16:2-OH (Hydroxyhexadecadienylcarnitine)	LC–MS/MS	0.02 ± 0.01
C16:1-OH (Hydroxyhexadecenoylcarnitine)	LC–MS/MS	0.021 ± 0.004
C16-OH (Hydroxyhexadecanoylcarnitine)	LC–MS/MS	0.011 ± 0.003
C18:2 (Octadecadienylcarnitine)	LC–MS/MS	0.006 ± 0.001
C18:1 (Octadecenoylcarnitine)	LC–MS/MS	0.011 ± 0.002
C18 (Octadecanoylcarnitine)	LC–MS/MS	0.008 ± 0.001
C18:1-OH (Hydroxyoctadecenoylcarnitine)	LC–MS/MS	0.011 ± 0.002
**TRACE ELEMENTAL COMPOUNDS**		
Lithium	ICP–MS	2 ± 1
Boron	ICP–MS	47 ± 28
Sodium	ICP–MS	25588 ± 4753
Magnesium	ICP–MS	10 ± 7
Phosphorus	ICP–MS	32820 ± 6825
Potassium	ICP–MS	46154 ± 8295
Calcium	ICP–MS	17 ± 8
Titanium	ICP–MS	2.2 ± 0.4
Cobalt	ICP–MS	0.8 ± 0.2
Copper	ICP–MS	27 ± 19
Zinc	ICP–MS	39 ± 60
Rubidium	ICP–MS	80 ± 16
Molybdenum	ICP–MS	3 ± 1
Cesium	ICP–MS	0.02 ± 0.01
Lead	ICP–MS	0.019 ± 0.003

[a] Miles et al., 2015 [59].

Table S4. List of testis metabolites along with their measured concentrations and their standard deviations (as measured in nmol/g).

Metabolite	Platform	Concentration	Literature value
**WATER-SOLUBLE COMPOUNDS**			
AMINO ACIDS			
Alanine *	LC–MS/MS & NMR	1400 ± 294	880–1200 [a]
Arginine	LC–MS/MS	51 ± 12	200–460 [a]
Asparagine	LC–MS/MS	48 ± 11	
Aspartate *	LC–MS/MS & NMR	597 ± 215	310–550 [a]
Beta-alanine	NMR	48 ± 13	
Citrulline	LC–MS/MS	19 ± 7	
Creatine	LC–MS/MS & NMR	7553 ± 1850	
Glutamate	LC–MS/MS & NMR	3270 ± 702	1950–2430 [a]
Glutamine	LC–MS/MS & NMR	1517 ± 338	
Glutathione	NMR	1453 ± 257	
Glycine *	LC–MS/MS & NMR	1247 ± 251	930–1250 [a]
Histidine	LC–MS/MS	61 ± 13	20 [b]
Isoleucine	LC–MS/MS & NMR	79 ± 14	30 [b]
Leucine	LC–MS/MS & NMR	138 ± 23	40 [b]
Lysine *	LC–MS/MS	64 ± 15	40 [b], 120–200 [a]
Methionine	LC–MS/MS & NMR	26 ± 6	
Ornithine	LC–MS/MS	10 ± 3	
Phenylalanine	LC–MS/MS & NMR	56 ± 12	
Proline	LC–MS/MS	191 ± 40	
Serine *	LC–MS/MS	272 ± 58	230 [b], 630–830 [a]
Threonine	LC–MS/MS & NMR	232 ± 51	140 [b]
Tryptophan	LC–MS/MS & NMR	18 ± 4	
Tyrosine	LC–MS/MS & NMR	43 ± 9	
Valine	LC–MS/MS & NMR	197 ± 37	50 [b]
BIOGENIC AMINES			
Acetyl-ornithine	LC–MS/MS	7 ± 3	
Asymmetric-dimethylarginine	LC–MS/MS	0.8 ± 0.2	
Carnosine	LC–MS/MS	12 ± 3	
Creatinine	LC–MS/MS & NMR	123 ± 26	
Kynurenine	LC–MS/MS	0.9 ± 0.4	
Methionine-sulfoxide	LC–MS/MS	1 ± 0.4	
Methylhistidine	LC–MS/MS	19 ± 4	
O-phosphoethanolamine	NMR	6934 ± 1186	
Putrescine	LC–MS/MS	11 ± 3	
Sarcosine	LC–MS/MS & NMR	5 ± 2	
Spermidine	LC–MS/MS	0.4 ± 0.2	
Taurine	LC–MS/MS & NMR	1510 ± 296	
Total-dimethylarginine	LC–MS/MS	1.9 ± 0.4	
Trans-hydroxyproline	LC–MS/MS	43 ± 12	

Trimethylamine N-oxide	LC–MS/MS	11 ± 5
CARBOHYDRATES		
Glucose	LC–MS/MS & NMR	149 ± 66
UDP-galactose	NMR	53 ± 10
UDP-glucose	NMR	126 ± 32
UDP-N-acetylglucosamine	NMR	268 ± 40
ORGANIC ACIDS		
3-hydroxybutyrate	NMR	101 ± 23
Acetate	NMR	88 ± 19
Alpha-aminoadipate	LC–MS/MS	98 ± 39
Ascorbate (Vitamin C)	NMR	1576 ± 363
Formate	NMR	823 ± 240
Fumarate	NMR	27 ± 8
Inosinate (IMP)	NMR	97 ± 19
Lactate	NMR	7702 ± 1649
Nicotinurate	NMR	38 ± 11
Pyruvate	NMR	11 ± 3
Succinate	NMR	340 ± 76
MISCELANEOUS		
Adenosine	NMR	150 ± 114
Betaine	LC–MS/MS & NMR	564 ± 134
Choline	LC–MS/MS & NMR	177 ± 71
Ethanol	NMR	125 ± 111
Glycerol	NMR	380 ± 112
Hypoxanthine	NMR	176 ± 66
Inosine	NMR	225 ± 95
Myo-inositol	NMR	7193 ± 1232
NAD+	NMR	151 ± 22
NADH	NMR	21 ± 8
NADP+	NMR	12 ± 3
O-phosphocholine	NMR	1058 ± 234
sn-Glycero-3-phosphocholine	NMR	702 ± 121
Uridine monophosphate (UMP)	NMR	56 ± 11
Uridine	NMR	156 ± 36
**LIPID-LIKE COMPOUNDS**		
PHOSPHATIDYLCHOLINES, ACYL-ALKYL		
PC ae (36:0)	LC–MS/MS	0.9 ± 0.2
PC ae (40:6)	LC–MS/MS	0.6 ± 0.1
PHOSPHATIDYLCHOLINES, DIACYL		
PC aa (32:2)	LC–MS/MS	0.5 ± 0.1
PC aa (36:6)	LC–MS/MS	0.4 ± 0.1
PC aa (36:0)	LC–MS/MS	15 ± 3
PC aa (38:6)	LC–MS/MS	21 ± 5
PC aa (38:0)	LC–MS/MS	0.5 ± 0.1
PC aa (40:6)	LC–MS/MS	2.7 ± 0.4

PC aa (40:2)	LC–MS/MS	0.12 ± 0.02
PC aa (40:1)	LC–MS/MS	0.07 ± 0.01
LYSOPHOSPHATIDYLCHOLINES, ACYL C		
LysoPC(14:0)	LC–MS/MS	0.09 ± 0.01
LysoPC(16:1)	LC–MS/MS	0.03 ± 0.01
LysoPC(16:0)	LC–MS/MS	0.7 ± 0.1
LysoPC(17:0)	LC–MS/MS	0.03 ± 0.01
LysoPC(18:2)	LC–MS/MS	0.41 ± 0.13
LysoPC(18:1)	LC–MS/MS	0.4 ± 0.1
LysoPC(18:0)	LC–MS/MS	0.16 ± 0.03
LysoPC(20:4)	LC–MS/MS	0.2 ± 0.1
LysoPC(20:3)	LC–MS/MS	0.04 ± 0.01
LysoPC(24:0)	LC–MS/MS	0.04 ± 0.01
LysoPC(26:1)	LC–MS/MS	0.04 ± 0.01
LysoPC(26:0)	LC–MS/MS	0.08 ± 0.01
LysoPC(28:1)	LC–MS/MS	0.06 ± 0.01
LysoPC(28:0)	LC–MS/MS	0.08 ± 0.01
SPHINGOMYELINS		
SM(16:1)	LC–MS/MS	0.6 ± 0.1
SM(16:0)	LC–MS/MS	42 ± 6
SM(18:1)	LC–MS/MS	0.5 ± 0.1
SM(18:0)	LC–MS/MS	5 ± 1
SM(20:2)	LC–MS/MS	0.3 ± 0.1
HYDROXYSPHINGOMYELINS		
SM(14:1(OH))	LC–MS/MS	1.3 ± 0.2
SM(16:1(OH))	LC–MS/MS	1.8 ± 0.3
SM(22:2(OH))	LC–MS/MS	1.1 ± 0.2
SM(22:1(OH))	LC–MS/MS	1.3 ± 0.3
SM(24:1(OH))	LC–MS/MS	0.25 ± 0.04
ACYLCARNITINES		
C0 (Carnitine)	LC–MS/MS	20 ± 6
C2 (Acetylcarnitine)	LC–MS/MS	40 ± 11
C3:1 (Propenoylcarnitine)	LC–MS/MS	0.77 ± 0.13
C3 (Propionylcarnitine)	LC–MS/MS	0.6 ± 0.2
C4:1 (Butenylcarnitine)	LC–MS/MS	0.02 ± 0.01
C4 (Butyrylcarnitine)	LC–MS/MS	2.8 ± 0.7
C3-OH (Hydroxypropionylcarnitine)	LC–MS/MS	0.06 ± 0.01
C5:1 (Tiglylcarnitine)	LC–MS/MS	0.021 ± 0.003
C5 (Valerylcarnitine)	LC–MS/MS	0.27 ± 0.09
C4-OH (C3-DC) (Hydroxybutyrylcarnitine)	LC–MS/MS	0.16 ± 0.04
C6:1 (Hexenoylcarnitine)	LC–MS/MS	0.03 ± 0.01
C6 (C4:1-DC) (Hexanoylcarnitine)	LC–MS/MS	0.21 ± 0.06
C5-OH (C3-DC-M) (hydroxyvalerylcarnitine)	LC–MS/MS	0.08 ± 0.02
C5:1-DC (Glutaconylcarnitine)	LC–MS/MS	0.021 ± 0.004
C5-DC (C6-OH)(Glutarylcarnitine)	LC–MS/MS	0.021 ± 0.004

C8 (Octanoylcarnitine)	LC–MS/MS	0.06 ± 0.01
C5-M-DC (methylglutarylcarnitine)	LC–MS/MS	0.016 ± 0.003
C9 (Nonaylcarnitine)	LC–MS/MS	0.014 ± 0.002
C7-DC (Pimelylcarnitine)	LC–MS/MS	0.1 ± 0.1
C10:2 (Decadienylcarnitine)	LC–MS/MS	0.05 ± 0.01
C10:1 (Decenoylcarnitine)	LC–MS/MS	0.27 ± 0.04
C10 (Decanoylcarnitine)	LC–MS/MS	0.06 ± 0.01
C12:1 (Dodecenoylcarnitine)	LC–MS/MS	0.13 ± 0.01
C12 (Dodecanoylcarnitine)	LC–MS/MS	0.04 ± 0.01
C14:2 (Tetradecadienylcarnitine)	LC–MS/MS	0.011 ± 0.002
C14:1 (Tetradecenoylcarnitine)	LC–MS/MS	0.011 ± 0.003
C14 (Tetradecanoylcarnitine)	LC–MS/MS	0.02 ± 0.01
C12-DC (Dodecanedioylcarnitine)	LC–MS/MS	0.013 ± 0.002
C14:2-OH (Hydroxytetradecadienylcarnitine)	LC–MS/MS	0.007 ± 0.001
C14:1-OH (Hydroxytetradecenoylcarnitine)	LC–MS/MS	0.009 ± 0.002
C16:2 (Hexadecadienylcarnitine)	LC–MS/MS	0.006 ± 0.001
C16:1 (Hexadecenoylcarnitine)	LC–MS/MS	0.032 ± 0.003
C16 (Hexadecanoylcarnitine)	LC–MS/MS	0.02 ± 0.01
C16:2-OH (Hydroxyhexadecadienylcarnitine)	LC–MS/MS	0.007 ± 0.001
C16:1-OH (Hydroxyhexadecenoylcarnitine)	LC–MS/MS	0.011 ± 0.001
C16-OH (Hydroxyhexadecanoylcarnitine)	LC–MS/MS	0.009 ± 0.001
C18:2 (Octadecadienylcarnitine)	LC–MS/MS	0.004 ± 0.001
C18:1 (Octadecenoylcarnitine)	LC–MS/MS	0.009 ± 0.002
C18 (Octadecanoylcarnitine)	LC–MS/MS	0.02 ± 0.01
C18:1-OH (Hydroxyoctadecenoylcarnitine)	LC–MS/MS	0.009 ± 0.002
**TRACE ELEMENTAL COMPOUNDS**		
Lithium	ICP–MS	19 ± 2
Boron	ICP–MS	31 ± 11
Sodium	ICP–MS	35578 ± 6600
Magnesium	ICP–MS	70 ± 23
Phosphorus	ICP–MS	15602 ± 2618
Potassium	ICP–MS	48919 ± 8298
Calcium	ICP–MS	13 ± 4
Titanium	ICP–MS	1 ± 0.2
Cobalt	ICP–MS	0.05 ± 0.01
Copper	ICP–MS	0.7 ± 0.1
Zinc	ICP–MS	4 ± 1
Rubidium	ICP–MS	49 ± 9
Molybdenum	ICP–MS	0.06 ± 0.01
Cesium	ICP–MS	0.02 ± 0.01
Thallium	ICP–MS	0.0014 ± 0.0003
Lead	ICP–MS	0.014 ± 0.003

[*] Compounds that exhibited good agreement with literature values; [a] Brown-Woodman and White, 1974 [32]; [b] Sexton et al., 1971 [54].
